# Two-input protein logic gate for computation in living cells

**DOI:** 10.1038/s41467-021-26937-x

**Published:** 2021-11-16

**Authors:** Yashavantha L. Vishweshwaraiah, Jiaxing Chen, Venkat R. Chirasani, Erdem D. Tabdanov, Nikolay V. Dokholyan

**Affiliations:** 1 Departments of Pharmacology, Penn State College of Medicine, Hershey, PA 17033-0850 USA; 2 Departments of Biochemistry & Molecular Biology, Penn State College of Medicine, Hershey, PA 17033-0850 USA; 3 Department of Chemistry, Pennsylvania State University, University Park, PA, 16802 USA; 4 Department of Biomedical Engineering, Pennsylvania State University, University Park, PA, 16802 USA

**Keywords:** Synthetic biology, Synthetic biology

## Abstract

Advances in protein design have brought us within reach of developing a nanoscale programming language, in which molecules serve as operands and their conformational states function as logic gates with precise input and output behaviors. Combining these nanoscale computing agents into larger molecules and molecular complexes will allow us to write and execute “code”. Here, in an important step toward this goal, we report an engineered, single protein design that is allosterically regulated to function as a ‘two-input logic OR gate’. Our system is based on chemo- and optogenetic regulation of focal adhesion kinase. In the engineered FAK, all of FAK domain architecture is retained and key intramolecular interactions between the kinase and the FERM domains are externally controlled through a rapamycin-inducible uniRapR module in the kinase domain and a light-inducible LOV2 module in the FERM domain. Orthogonal regulation of protein function was possible using the chemo- and optogenetic switches. We demonstrate that dynamic FAK activation profoundly increased cell multiaxial complexity in the fibrous extracellular matrix microenvironment and decreased cell motility. This work provides proof-of-principle for fine multimodal control of protein function and paves the way for construction of complex nanoscale computing agents.

## Abstract (web summary)

Traditional synthetic biology tools operate by complex re-programming of DNA, requiring significant amount of ‘nucleotide-based code’ to implement instructions that are transcribed at the protein level. Here the authors demonstrate the direct regulation of cellular phenotype at the single-protein level by creating a two-input logic gate for biological computation using ‘allosteric wiring’.

## Introduction

Cellular programming involves embedding of instructions within natural cellular constituents (e.g., DNA, RNA, or protein) to control phenotype^1^. The process hijacks one or more components and alters their function using external (e.g., organic or inorganic molecules or light)^2^ or internal controls^3^. Cellular programming offers unparalleled opportunities in biotechnology and medicine, and its most intriguing features are the abilities to piggyback on natural processes and to incorporate evolutionary pressure into the syntax. Most of the developments in the field of synthetic biology have focused on reprogramming DNA sequence^4^. The advantage of using DNA as a “programming language” is its simplicity: control is established by regulating expression of a particular gene, and, hence, the activation of the encoded protein. Such simplicity comes with the disadvantage of a “heavy” program: reprogramming requires that many kilobases of nucleotides be re-written or added in order to control activity of a single protein. Alternatively, we can directly control the function of a protein by “rewiring” its structure without the overhead of gene expression machinery. The challenge in this case is that protein 3D structure and dynamics need to be reprogrammed. Robust input/output control has been reported in various systems using protein engineering approaches^2,5–7^, but to create a program, logic gates must be incorporated into the circuit.

Several attempts have been made to create the protein logic gates, but most involve either indirect control or are multi-protein systems^8–11^. A directly regulated, single protein design offers simplicity, tight regulation, and targeted control. Here, we develop a single protein system directly integrated with two orthogonal, two-input logic gates as regulatory switches. We combined chemogenetic^7,12,13^ and optogenetic approaches^14^ to establish two-input control over focal adhesion kinase (FAK). FAK is a highly conserved non-receptor tyrosine kinase present in high abundance in focal adhesions that functions in regulation of the cytoskeleton^15^. FAK expression is abnormally high in certain types of cancer; FAK blocks apoptotic signaling and enhances invasiveness^16^. To create a logic gate to regulate FAK functions, we allosterically embedded two regulatory domains, the rapamycin-inducible uniRapR domain^12^ and a light-oxygen-voltage-sensing LOV2 domain^17^, within FAK. This chemo-opto controlled protein, *ChOp*-FAK, functionally serves as the digital “two-input logic OR gate”. Our study provides proof-of-principle that a single protein can be programmed as a logic gate by allosteric wiring of two inputs to a functional site, which serves as an output. By expression of our designed protein in cells, we demonstrated that in the fibrous extracellular matrix (ECM) microenvironment, activated FAK promotes adhesion, higher-order spatial dimensionality, and architectural complexity, reducing cellular motility.

## Results

### Design of *ChOp*-FAK

**Fig. 1:**
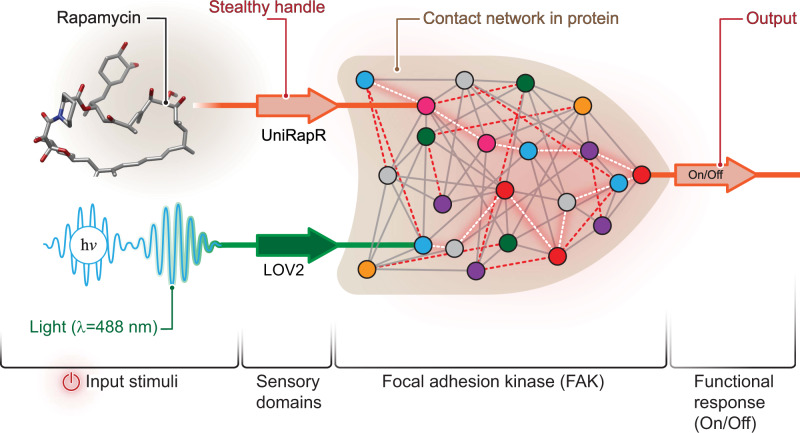
*ChOp*-FAK resembles digital two-input logic OR gate. *Chop*-FAK is allosterically regulated by inserted sensor domains uniRapR and LOV2, which serve as input response elements. Rapamycin and light are the input signals for uniRapR and LOV2, respectively. From the sensor domains signals propagate through the amino acid core network (shown as contact network) of the protein. The output is FAK activation.

To develop a protein that functions as a two-input logic OR gate, we engineered a dual-regulated kinase *ChOp*-FAK with two rationally incorporated regulatory sensor modules (Fig. 1). The chemogenetic (*Ch*) module is uniRapR, which activates the protein in response to its binding partner rapamycin (Supplementary Fig. 1a). The second module is the optogenetic (*Op*) LOV2 domain, which inactivates the protein in response to blue light (Supplementary Fig. 1b). Rapamycin and light serve as the input signals, and kinase function is the output signal. The FAK structure includes an N-terminal FERM domain, central kinase domain, and C-terminal FAT domain. The FERM and kinase domains play direct roles in the catalytic regulation of FAK^18^. Therefore, to build *ChOp*-FAK and to enable dual regulation, we rationally introduced chemo and opto orthogonal switches into the kinase domain and FERM domain, respectively.

### Design, preparation, and validation of *Ch*-FAK

**Fig. 2:**
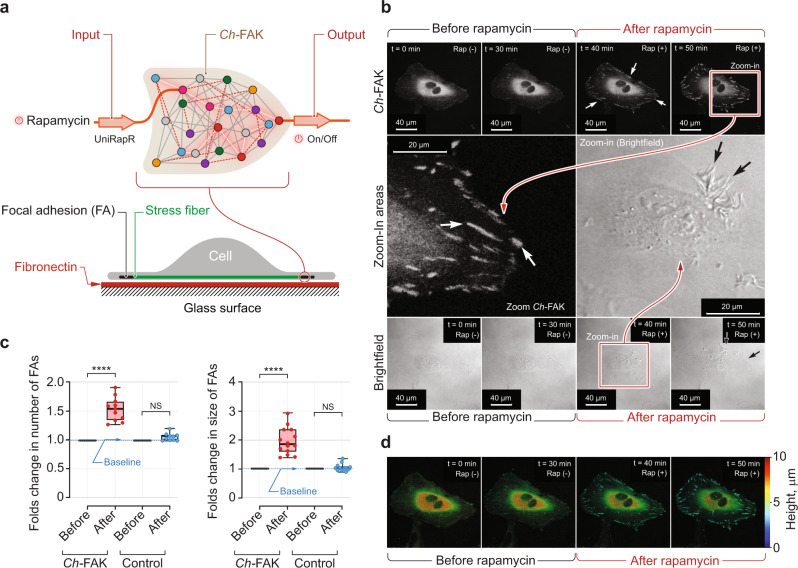
Design and validation of *Ch-*FAK module. **a** Top, schematic of *Ch*-FAK. 50 nM rapamycin is the input signal for *Ch*-FAK. Output is measured as *Ch*-FAK activation. Bottom, schematic of a cell plated on fibronectin-coated glass surface showing stress fibers, focal adhesions and the location of activated *Ch*-FAK. **b** Top, time-lapse fluorescent imaging data for the mCherry-tagged *Ch*-FAK demonstrating rapamycin-induced activation in HeLa cells. 50 nm rapamycin was added at 30th min to induce the activation. Enlarged, late focal adhesions are indicated by arrows. Bottom, time-lapse bright-field images of HeLa cells that express *Ch*-FAK before and after addition of rapamycin. Dorsal ruffles are indicated by arrows. Center, zoomed-in regions of indicated images. **c** Normalized quantification of average size and total number of focal adhesions before and after the rapamycin addition. Control is kinase-dead mutant of FAK. Data represent box plots and individual data points. Box plots show the median (center line), first and third quartiles (box edges), while the whiskers going from each quartile to the minimum or maximum. *n* = 11 cells for total focal adhesions (FAs) and *n* = 14 cells for average size of FAs from 3 independent experiments; ^****^P = 2.6 × 10^−8^ for number of FAs, ^****^P = 2.7 × 10^−8^ for size of FAs in *Ch*-FAK. *P* = 0.872 for number of FAs and *P* = 0.1464 for FAs size in control conditions calculated by unpaired two-tailed Student’s *t*-test. NS, not significant. **d** Depth analysis for the time-lapse imaging data for rapamycin-induced activation in *Ch*-FAK-expressing HeLa cells. Analysis was performed by measuring the distance between the glass surface and each optical planes of the cell. Distances are indicated by a color scale. Scale bar, 40 μm. FAs indicates focal adhesions. Source data are provided as a Source Data file.

We first built the chemogenetically controlled FAK (*Ch*-FAK) by integrating the rapamycin-binding uniRapR domain into the kinase domain (Fig. 2a). Kinases containing the uniRapR domain remain catalytically inactive until the domain binds rapamycin^7,19^. Rapamycin stabilizes the uniRapR domain structure, which, in turn, results in activation of the kinase. Our previous study demonstrated that FAK activity could be allosterically regulated by the insertion of a regulatory domain into the kinase domain^7^. Hence, we introduced the uniRapR domain into the previously identified allosteric site on the kinase domain of FAK. Short GPG linkers were introduced on both the sides of the uniRapR domain to optimize regulation (Supplementary Table 2). We performed DMD simulations for *Ch*-FAK in presence and absence of rapamycin. Rapamycin-bound structure displayed an open conformation, corresponding to the active conformation of FAK, whereas simulations without rapamycin showed a closed confirmation of the protein, suggesting the inactive form (Supplementary Fig. 2). We also incorporated two reported mutations, Y180A and M183A, into *Ch*-FAK to rule out the possibility of FAK activation by endogenous upstream factors^7^.

We expressed *Ch*-FAK in HeLa cells, and cultured these cells on a coverslip in a glass-bottomed dish. Addition of 50 nM rapamycin to the *Ch*-FAK-expressing cells increased the membrane dynamics of the cells as shown by imaging of live cells over time (Fig. 2b). After rapamycin addition, dorsal ruffles formed, and we observed localization of FAK within these membrane ruffles (Supplementary Fig. 3). These results are in agreement with our previous findings^7^. The dorsal ruffle phenotype was observed in only 10 of 30 cells, therefore we looked for alternate output signals.

We observed that rapamycin treatment promoted translocation of *Ch-*FAK from the cytoplasmic regions to focal adhesions resulting in their enlargement (Fig. 2b, Supplementary Fig. 4, and Supplementary Movie 1). The conversion from small, early focal adhesions to large, late focal adhesions was very rapid, occurring about 10 min post-rapamycin treatment. Quantitative analysis showed an approximately 1.6-fold increase (*P* < 0.0001 by unpaired Student’s *t*-test) in the total number of focal adhesions per cell post-rapamycin addition (Fig. 2c). Importantly, there was an approximately 1.9-fold increase (*P* < 0.0001 by unpaired Student’s *t*-test) in the average size of the focal adhesions upon rapamycin treatment (Fig. 2c). In cells that expressed the control construct, *Ch*-FAK-YM-KD, which is a mutant of FAK lacking catalytic activity, we did not observe any significant changes in the focal adhesions upon rapamycin addition (Fig. 2c). The majority of the large, late focal adhesions were located on the side of the cells next to the culture plate (Fig. 2d). FAK and Paxillin phosphorylation studies suggested that *Ch*-FAK’s catalytic activity was activated upon rapamycin treatment (Supplementary Fig. 5).

**Fig. 3:**
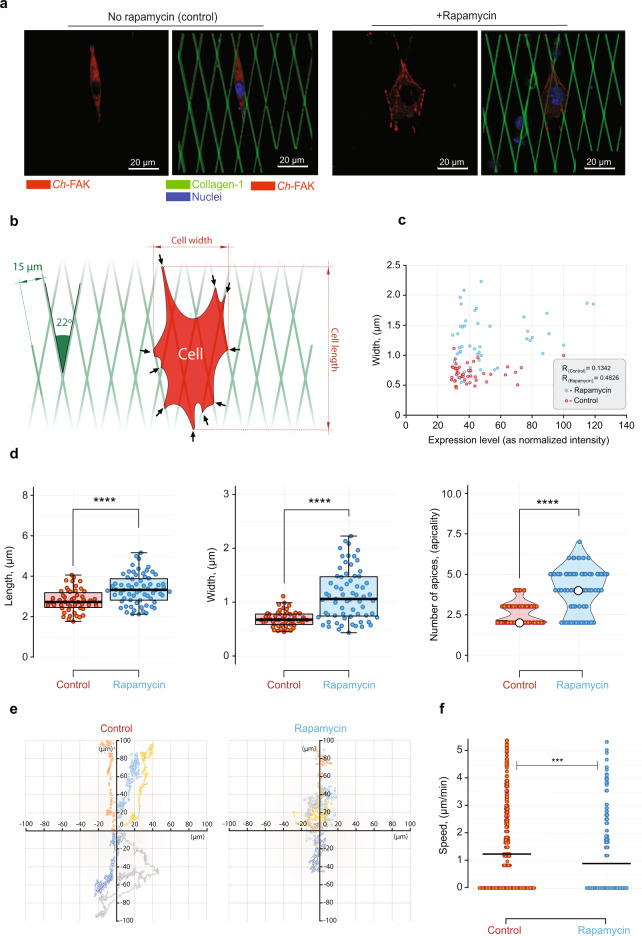
Cell motility and morphological response to FAK activation on the structurally standardized biomimetic collagen type-1 fibers. **a** Representative cell fluorescent micrographs for *Ch*-FAK-expressing *FAK*^−/−^ cells with (right) and without (left) rapamycin treatment on 2D collagen type-1 micropatterns. Red fluorescence indicates *Ch*-FAK. Scale bar, 20 μm. **b** Schematic of the cell morphometric analysis. Arrows indicate apices (apicality) of the cell. **c** Width of *Ch*-FAK-transfected *FAK*^−/−^ fibroblasts with and without rapamycin treatment versus *Ch*-FAK expression levels. Data represent scatter plot, *n* = 50 cells for controls and *n* = 67 cells for rapamycin-treated conditions from 3 independent experiments. **d** Length, width, and apicality of *Ch*-FAK-transfected *FAK*^−/−^ fibroblasts with and without rapamycin treatment. Data represent box plots, violin plots and individual data points. Data represent box plots and individual data points. Box plots show the median (center line), first and third quartiles (box edges), while the whiskers going from each quartile to the minimum or maximum. *n* = 50 cells for controls and *n* = 67 cells for rapamycin-treated conditions from 3 independent experiments. ^****^P = 1.5 × 10^−5^ for length, ^****^P = 3.6 × 10^−9^ for width, and ^****^P = 4.7 × 10^−11^ for apicality by unpaired two-tailed Student’s *t*-test. **e** Migration tracks of rapamycin-treated and untreated *Ch*-FAK-transfected MDA-MB-231 cells along the collagen fibers. Data represent migration tracks, *n* = 4 cells for control and rapamycin-treated conditions. **f** Migration speeds of the rapamycin-treated and untreated *Ch*-FAK-transfected MDA-MB-231 cells along the collagen fibers on 2D collagen type-1 micropatterns. *n* = 4 cells for control and rapamycin-treated conditions; ^***^P = 0.001 by unpaired two-tailed Student’s *t*-test. Source data are provided as a Source Data file.

Previous reports have implicated FAK in cell motility and spreading^20^. To test such effects, *FAK*^*−/−*^ fibroblasts were cultured on an architecturally standardized microenvironmental platform using 2D biomimetic collagen type-1 fibers as micropatterns^21^. *Ch*-FAK-transfected *FAK*^*−/−*^ fibroblasts exhibited a spindle morphology (Fig. 3a, left and Supplementary Fig. 6, left), very similar to wild-type fibroblast morphology is observed in 3D collagen ECM^22^. Upon treatment with rapamycin, there was a change from uniaxial spindle organization toward complex multiaxial cell architectures indicating a higher responsiveness to the cell microenvironments (Fig. 3a, right and Supplementary Fig. 6, right). These changes were evaluated by standardized cell morphometric analysis (Fig. 3b). *Ch*-FAK activation resulted in change in cell morphometric organization as indicated by increases in lengths, widths, and apicality along the rhomboid anisotropic biomimetic collagen “fibers” (Fig. 3c, d). We did not observe any morphological changes in *Ch*-FAK non-transfected cells upon rapamycin treatment (Supplementary Fig. 7). We also evaluated effect of *Ch*-FAK on cell motility and speed in MDA-MB-231 cells (Supplementary Movies 2 and 3). Treatment of these cells with rapamycin significantly reduced motility compared to untreated cells (Fig. 3e, f). Thus, we reasoned that *Ch*-FAK activation increases cellular adhesion and architectural complexity, which decreases cell motility.

**Fig. 4:**
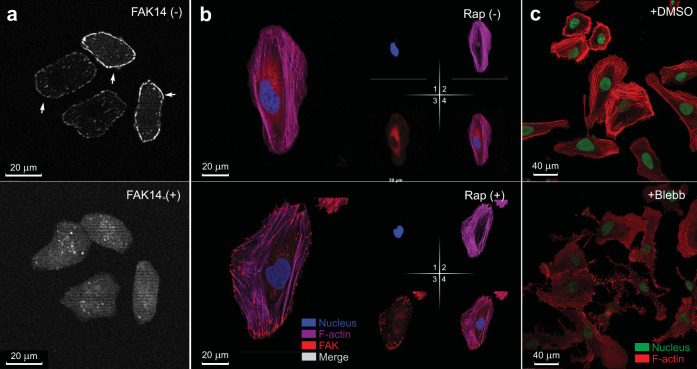
FAK inhibition blocks effects of rapamycin on *Ch*-FAK-expressing *FAK*^*−/−*^ fibroblasts. **a** Images of rapamycin-treated *Ch*-FAK-expressing *FAK*^−/−^ fibroblasts treated with control (DMSO) and FAK inhibitor 14. Top panel shows the *Ch*-FAK-activated *FAK*^*−/−*^ cells treated with control. Arrows indicate focal adhesions. Bottom panel shows same set of cells treated with FAK inhibitor 14. Scale bar, 20 μm. **b** Images of *Ch-*FAK-expressing *FAK*^−/−^ fibroblasts without (top) and with (bottom) rapamycin stained with phalloidin to reveal stress fibers (magenta). Large focal adhesions formed by the activation of *Ch*-FAK are visible as red clusters (mCherry fluorescence) at the ends of stress fibers in bottom panel. Nuclei were stained with Hoechst (blue). Individual channels are shown to the right of the merged images. Scale bar, 20 μm. **c** Images of rapamycin-treated *Ch*-FAK-expressing *FAK*^−/−^ fibroblasts treated with control (DMSO) (top) or blebbistatin (bottom). Red indicates phalloidin stained stress fibers and green indicates Hoechst-stained nucleus. Scale bar, 40 μm.

To confirm the role of *Ch-*FAK activation in the formation of enlarged focal adhesions, we treated the *Ch-*FAK-transfected, rapamycin-treated *FAK*^−/−^ fibroblasts with FAK inhibitor 14 and performed live-cell imaging. FAK inhibitor 14 treatment resulted in rapid degradation of enlarged, late focal adhesions only in the *Ch-*FAK-transfected cells (Fig. 4a and Supplementary Figs. 8 and 9). Focal adhesion growth depends on stress fibers^23^, which are crucial for mechanotransduction^24^. To verify the role of stress fibers in the effects of rapamycin on *Ch-*FAK-transfected *FAK*^−/−^ fibroblasts, we stained the cells for phalloidin to visualize the stress fibers during the *Ch*-FAK activation process. In these cells, focal adhesions were visible as large clusters at the stress fiber termini, and activation of *Ch*-FAK altered the distribution of intracellular stress fibers (Fig. 4b). When we treated rapamycin-activated cells with 50 µM of blebbistatin, a myosin-II inhibitor, the central and peripheral stress fibers disintegrated and focal adhesions were disrupted within 20 min, and the cells underwent drastic changes in morphology (Fig. 4c and Supplementary Fig. 10). These data demonstrate that *Ch-*FAK activation is responsible for the stress fiber-mediated alterations in focal adhesions in the *Ch-*FAK-transfected cells.

### Design, preparation, and validation of *Op*-FAK

We next introduced the light-inducible LOV2 domain from *Avena sativa* into a loop of the FERM domain of FAK. The 10 Å spacing between the N and C termini of LOV2 is ideal as it results in minimal perturbation of the structure of the loop of the protein into which it is inserted^2^. Blue light exposure leads to the unfolding of the C-terminal Jα helix of LOV2. This light-induced conformational change in LOV2 leads to the distortion of the FAK and resulting in its inactivation.

**Fig. 5:**
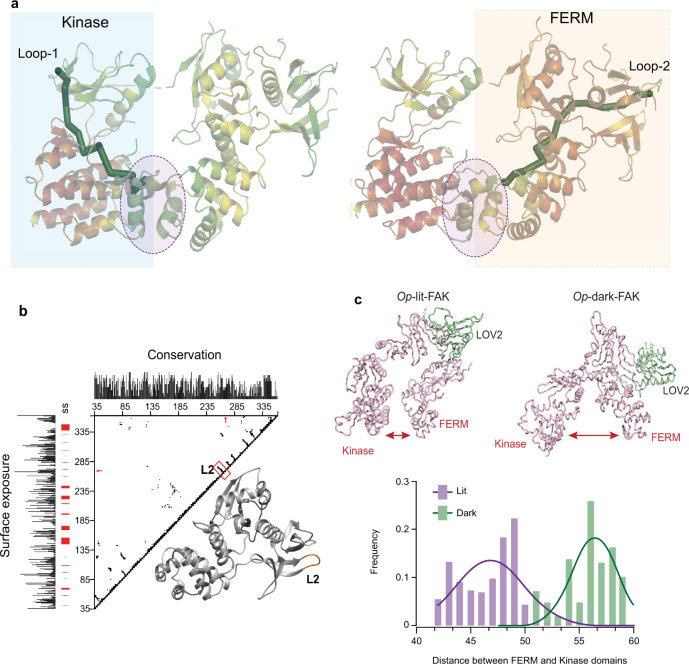
Computational identification of insertion site and DMD simulations. **a** Ribbon diagrams of FAK from the published crystal structure (PDB ID: 2JOJ). Loop 1 is the insertion site for uniRapR (left), and Loop 2 is the insertion site for LOV2 (bottom). Green paths represent signal propagation pathways from the insertion sites to the target region (circled). **b** Contact map computed from the published crystal structure (PDB ID: 2JOJ) for sequence conservation and surface exposure. Loop 2 (L2) has low sequence conservation and high surface exposure. Bottom figure demonstrates the β-hairpin loop (loop 2) within the FERM domain. **c** Top, snapshots from the DMD simulations showing conformations of *Op*-lit-FAK and *Op*-dark-FAK. The distance between the two domains in *Op*-dark-FAK is indicative of activation. Bottom, frequencies of structures with given distances between FERM and kinase domains in *Op*-lit-FAK and *Op*-dark-FAK quantified during DMD simulations. Source data are provided as a Source Data file.

**Fig. 6:**
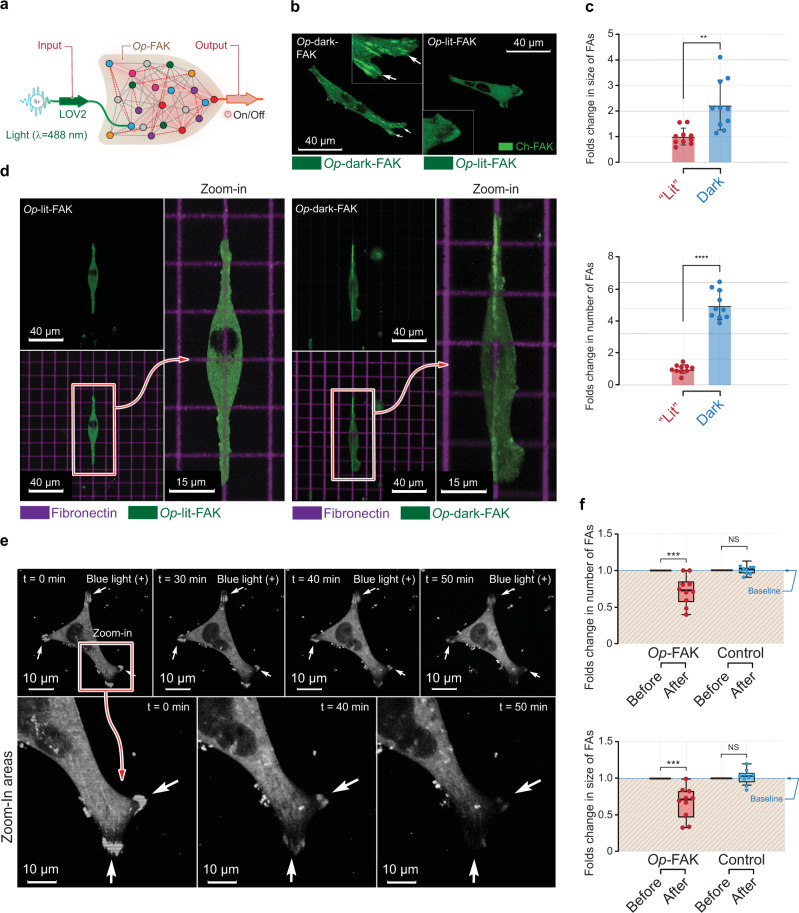
Activation of *Op*-FAK expressed in *FAK*^*−/−*^ cells is light sensitive. **a** Schematic representation of the *Op*-FAK. LOV2 domain is inserted into the FERM domain of FAK. Light is used as input signal. Dark condition activates FAK and light conditions inactivates FAK. **b** Images of cells transfected with *Op*-dark-FAK (left) and *Op*-lit-FAK (right) on a fibronectin-coated glass surface. Inset shows the cell edges with arrows highlighting focal adhesions. Scale bar, 40 μm. **c** Normalized quantification of average size and total number of focal adhesions in *Op*-dark-FAK- and *Op*-lit-FAK-expressing *FAK*^*−/−*^ cells on a fibronectin-coated glass surface. Data represent bar plots with mean±s.d. and individual data points, *n* = 10 cells for dark and lit mutants from 3 independent experiments; ^****^P = 6.2 × 10^−11^ for number of focal adhesion (FAs), ^**^P = 0.0014 for size of FAs in mutants calculated by unpaired two-tailed Student’s *t*-test. NS, not significant. **d** Images of *Op*-lit-FAK- (left) and *Op*-dark-FAK-expressing (right) *FAK*^*−/−*^ cells on PAA gels printed with fluorescently labeled fibronectin grids. Zoomed-in images show the well-formed focal adhesions along the grid lines for *Op*-dark-FAK. Scale bar, 40 μm; zoomed-in images, 14 μm. **e** Top, time-lapse images of *FAK*^−/−^ fibroblasts that express *Op*-FAK exposed to light for the indicated periods of time. Bottom, magnification of region boxed in the upper left panel as a function of time in response to blue light (488 nm). Arrows indicate focal adhesions. Scale bar, 40 μm. **f** Normalized quantification of average size and total number of focal adhesions during light-induced inactivation of *FAK*^*−/−*^ cells that express *Op*-FAK or *Op*-dark-FAK (control). FAs indicates focal adhesions. Data represent box plots and individual data points. Data represent box plots and individual data points. Box plots show the median (center line), first and third quartiles (box edges), while the whiskers going from each quartile to the minimum or maximum. *n* = 10 cells for total FAs and average size of FAs from 3 independent experiments; ^***^P = 0.0003 for number of FAs, ^***^P = 0.0001 for size of FAs in *Op*-FAK. *P* = 0.7737 for number of FAs and *P* = 0.4617 for FAs size in control conditions calculated by unpaired two-tailed Student’s *t*-test. NS, not significant. Source data are provided as a Source Data file.

To identify regions of the FERM domain that could be connected to LOV2 to result in allosteric control, we mapped the allosteric connectivity between the loops and target region using *Ohm* (Fig. 5a). We also considered three other parameters: sequence conservation, surface exposure, and loop tightness (Fig. 5b). Based on these criteria, loop 2, between S264 and S265, was selected as a potential insertion site. We used DMD simulations to analyze conformations of *Op*-dark-FAK and *Op*-lit-FAK, with mutants of LOV2 locked in the dark and light states, respectively, inserted at loop 2 (Fig. 5c). We observed a closed conformation in *Op*-lit-FAK, corresponding to an inactive conformation of FAK, whereas *Op*-dark-FAK had an open, active conformation. In design of *Op*-FAK, we also considered linkers of different lengths and sequences for connection of the LOV2 domain to FERM. A short GP linker was optimal (Fig. 6a and Supplementary Table 2).

We first expressed *Op*-dark-FAK and *Op*-lit-FAK in *FAK*^−/−^ fibroblasts and evaluated the responses. We observed large focal adhesions, similar to those observed upon expression of activated *Ch*-FAK, in *Op*-dark-FAK-transfected cells but not in *Op*-lit-FAK-transfected cells (Fig. 6b). Upon quantitation, we discovered that focal adhesions were approximately 2-fold larger in size (*P* < 0.01 by unpaired Student’s *t*-test) and that there were approximately 4.5-fold more focal adhesions (*P* < 0.0001 by unpaired Student’s *t*-test) in cells that expressed *Op*-dark-FAK than in those that expressed *Op*-lit-FAK (Fig. 6c). Moreover, on rigid PAA gels (*G*′ of 8.6 kPa) microprinted with fluorescent fibronectin grids to mimic the fibrous ECM, most *Op*-dark-FAK-transfected cells showed clear and well-formed focal adhesions along the fibronectin, whereas the *Op*-lit-FAK-transfected cells did not form focal adhesions (Fig. 6d).

We next tested cells that express the kinase with the wild-type LOV2 (*Op*-FAK). We performed live-cell imaging of the *Op*-FAK-transfected *FAK*^−/−^ fibroblasts for 30 min in the dark and then during 60 min of exposure to blue light. In the dark, cells had numerous, enlarged focal adhesions, similar to those formed by cells that express *Op*-dark-FAK. When we exposed the cells to blue light, the number and average size of the focal adhesions decreased by about 0.7 fold (*P* < 0.001 by unpaired Student’s *t*-test) within 35 min (Fig. 6e, f). As a control, we evaluated the effect of blue light on *Op*-dark-FAK-transfected cells but did not observe any significant changes in focal adhesions (Fig. 6f). Analysis of FAK and paxillin phosphorylation levels indicated that the *Op*-FAK’s catalytic activity was inhibited upon light irradiation (Supplementary Fig. 5).

Unlike the effects of *Ch*-FAK activation, which occurred very rapidly, inactivation of *Op*-FAK was a slow process. To improve the response, we tested the transfected cells under different conditions. Substrate stiffness influences the cellular responses^25^. Therefore, we evaluated the light response of the *Op*-FAK-transfected cells on a soft elastic hydrogel surface (*G*′ of 2.3 kPa). There was not a significant improvement in the inactivation kinetics (Supplementary Fig. 11). We also substituted rested collagen type-1 coating of the glass surface but the response rate did not differ from that on a fibronectin-coated surface (Supplementary Fig. 12). Even though activation/inactivation kinetics are important when designing optogenetic or chemogenetic tools, the kinetics of *Op*-FAK were sufficient for us to build and test the gating functions.

### Assembly and testing of *ChOp*-FAK

**Fig. 7:**
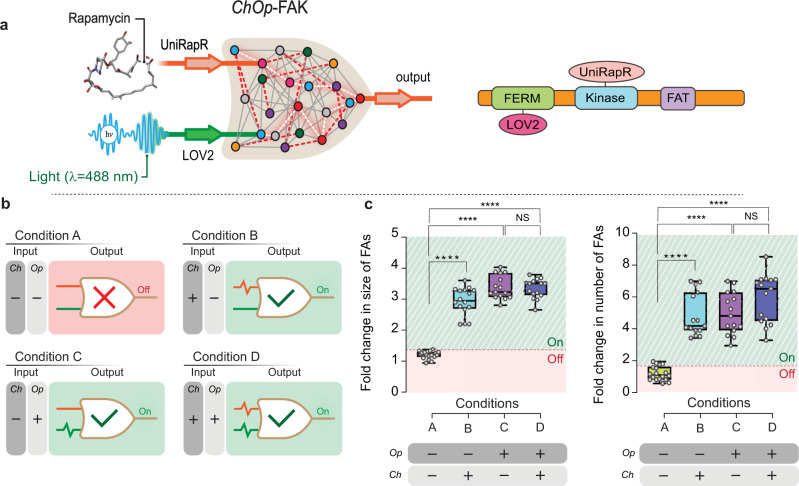
Design and functionality of *ChOp*-FAK. **a** Left, schematic representation of *ChOp*-FAK. Validated *Ch* and *Op* modules were assembled to construct *ChOp*-FAK. Right, domain organization of *ChOp*-FAK. **b** Combinations of tested input conditions and the respective outputs. For *Ch* input, *ChOp*-FAK-expressing *FAK*^*−/−*^ cells were treated with 50 nM rapamycin for 30 min. For Op input, dark and lit mutants of LOV2 were used to mimic the dark and light conditions, respectively. **c** Quantification of average size and total number of focal adhesions in *FAK*^*−/−*^ cells expressing linker optimized *ChOp*-FAK. Pink box indicates inactivation and green box indicates activation of FAK. Data were normalized to Condition A. Focal adhesion (FAs) indicates focal adhesions. Data represent box plots and individual data points. Data represent box plots and individual data points. Box plots show the median (center line), first and third quartiles (box edges), while the whiskers going from each quartile to the minimum or maximum. *n* = 18 cells for Condition A, *n* = 16 cells for Condition B, *n* = 15 cells for Conditions C and D from 3 independent experiments. For total number of FAs, ^****^P = 4.3 × 10^−12^ for A and B, ^****^P = 1.07 × 10^−12^ for A and C, ^****^P = 1.18 × 10^−13^ for A and D, *P* = 0.08 for C and D. For Average size of FAs, ^****^P = 1.2 × 10^−16^ for A and B, ^****^P = 7.25 × 10^−23^ for A and C, ^****^P = 5 × 10^−21^ for A and D, *P* = 0.768 for C and D conditions calculated by unpaired two-tailed Student’s *t*-test. NS, not significant. Source data are provided as a Source Data file.

To build our final design, we engineered FAK with both chemo- and optogenetically regulated domains with uniRapR and LOV2 designs inserted into the kinase domain and the FERM domain, respectively (Fig. 7a). For these experiments, we used the *ChOp*-lit-FAK and *ChOp*-dark-FAK constructs for two reasons. When we attempted to start in the light conditions, phototoxicity was observed because of the prolonged exposure. In the dark conditions, FAK was activated and no difference was observed upon rapamycin addition. We therefore subjected cells that expressed *ChOp*-lit-FAK and *ChOp*-dark-FAK to input signals and measured the corresponding output signals (Fig. 7b). Rapamycin was the activating input condition for the *Ch* module, and the dark- and lit-state mutations served as activated and inactivated states for the *Op* module. In *FAK*^*−/−*^ cells transfected with *ChOp*-lit-FAK not treated with rapamycin, we anticipated that the two “OFF” signals would maintain the kinase and FERM domains in close proximity ensuring a strong inactivation. Indeed, only the small, early focal adhesions were observed in these cells, and these foci were comparable to those in cells in which *Op*-lit-FAK was expressed, suggesting inactivation of FAK. We quantified the number and the average size of the focal adhesions, and we used these parameters to identify activating conditions (Fig. 7c, Condition A). Next, we treated cells that expressed *ChOp*-lit-FAK with rapamycin, the input signal for the *Ch* module. Upon addition of 50 nM rapamycin, there was not a significant increase in the average size or total number of the focal adhesions compared to *ChOp*-lit-FAK not treated with rapamycin (Supplementary Fig. 13). At 100 nM rapamycin concentration, the average size did not change significantly, but there was a significant increase in the total number of focal adhesions (*P* < 0.05 by unpaired Student’s *t*-test) (Supplementary Fig. 13b). At 150 nM rapamycin, we observed significant increases in the average size (*P* < 0.001 by unpaired Student’s *t*-test) and the total number of focal adhesions (*P* < 0.05 by unpaired Student’s *t*-test) (Supplementary Fig. 13b), indicative of activation of the *Ch* module (Supplementary Fig. 13b). However, the activation was not very robust. To optimize the *ChOp*-FAK design, we modulated the strength of input via redesign of linkers. We introduced several linkers (differing in length and sequence) into *ChOp*-FAK and screened them using western blot (Supplementary Tables 1 and 2 and Supplementary Fig. 14). From the screening, we picked the optimized design (with linker “G”) and continued the evaluation. The results indicated that performance of the optimized *ChOp*-FAK design improved drastically in “Condition B” with ~2.9-fold increase (*P* < 0.0001 by unpaired Student’s *t*-test) in the average size of focal adhesions and ~4-fold increase (*P* < 0.0001 by unpaired Student’s *t*-test) in total number of focal adhesions compared to cells that expressed *ChOp*-lit-FAK not treated with rapamycin (Fig. 7c, Condition B). We next evaluated *ChOp*-dark-FAK expressed in *FAK*^*−/−*^ cells in the absence and presence of rapamycin. In the absence of rapamycin, the average size of focal adhesions was 3.3-fold higher (*P* < 0.0001 by unpaired Student’s *t*-test) and the number of focal adhesions was about 4.5-fold higher (*P* < 0.0001 by unpaired Student’s *t*-test) than in cells that expressed *ChOp*-lit-FAK not treated with rapamycin (Fig. 7c, Condition C). This suggested a near-complete activation of *ChOp*-FAK. When rapamycin was added to the *ChOp*-dark-FAK-expressing cells, sizes and numbers of focal adhesions were similar to those in the absence of rapamycin (Fig. 7c, Condition D).

## Discussion

Here, we engineered a protein as a “two-input logic OR gate” by embedding two regulatory domains, uniRapR and LOV2, into the kinase FAK. Previously, we had engineered several opto- and chemo-kinases that enabled allosteric control of the kinase activity^2,7,13^. This allosteric regulation was highly specific and minimized interference from the active site and its binding partners^26^. Here, we applied similar design principles to build the gating function. Unlike tools of synthetic biology that require “rewriting” of DNA encoding many proteins, our approach aims at direct regulation of cellular phenotype at a single protein level.

Signals from the allosterically introduced orthogonal switches are propagated through independent pathways to the target site to achieve logical functions. We discovered that activation of *ChOp*-FAK was rapid, resulting in the formation of enlarged, late focal adhesions and increased dorsal ruffles and enhancing the adhesive capabilities of the cells, which decreased cellular motility. Activation also increased multiaxial orientation and higher order of spatial dimensionality in cells. The effects of the cell dimensionality have been studied from the perspective of the cell motility^27^, but little is known about correlative and causative links between FAK activity and cell structural complexity in the architecturally complex microenvironments. This system will be useful for further study of this phenomenon. The inactivation of *ChOp*-FAK was slow. We speculate that this is because the layered organization in focal adhesions might hinder the inactivation of FAK (Supplementary Fig. 15).

Our study demonstrates the response to input of a rationally designed nano-computing agent (NCA)^1^ as *ChOp*-FAK can function as logic OR gate. Proteins play vital roles in information processing in the cell as both input and output signals. Unlike traditional synthetic biology approaches, which are based on the rewiring of signaling pathways in cells, NCAs are autonomous single-protein devices. As NCAs could be used to monitor and regulate biological systems, they have potential for use in various biomedical and biotechnological applications such as disease diagnostics and drug delivery, control of metabolic flux, rewiring of cellular signaling, and context-based sensing of metals, pH, and temperature. We anticipate that in the near future these NCAs will operate in vivo within living organisms.

In summary, for the first time we created a two-input logic OR gate in a single protein through allosteric regulation by combining two orthogonal regulators. The architecture presented in this work can be extended to other proteins. We envision that this approach will pave the way for construction of more robust and complex NCAs with potential for biomedical and biotechnological applications.

## Methods

### Computational identification of insertion sites, molecular modeling, and discrete molecular dynamics simulations

We reconstructed the missing atoms and residues in the crystal structures of FAK (PDB ID: 2J0J)^18^ and LOV2 (PDB ID: 2V0U)^28^ using Modeller-9v14^29^. To mimic light-induced unfolding of Jα helix in LOV2, we applied repulsive potentials through short discrete molecular dynamics (DMD) simulations^30,31^. We energetically optimized all structural models through short DMD simulations performed at high temperature (0.7 kcal/mol·k_B_, where k_B_ is the Boltzmann constant). To identify optimal locations for insertion of LOV2 into FAK, we calculated the solvent accessible surface area (SASA) of FAK and identified surface exposure loops with SASA ≥ 40 Å^28^; this type of approach was used previously^2^. The β-hairpin loop between β6 and β7 in the FERM domain had an optimal SASA for LOV2 insertion. We used the *Ohm* server^32^ to determine whether the identified insertion region is allosterically connected to the target site. We excised the S264–S265 peptide bond and inserted LOV2 with the Jα helix either in a folded or unfolded conformation to represent dark and lit states, respectively. We employed Modeler-9v14^29^ to model chimeric FAK-LOV2 complex structures. For each model, we generated 40 candidates and selected the model with the least modeler objective function. When generating the models, we imposed restraints on FAK to avoid conformational changes due to LOV2 insertion. We initially optimized the modeled chimeric structures using Chiron^33^ and Gaia^34^ and subsequently employed constant-temperature, all-atom DMD simulations at 0.5 kcal/mol·k_B_. The inserted LOV2 domain remained folded as the employed DMD simulation temperature was less than protein folding transition temperature. We optimized the relative orientation of the LOV2 domain with respect to FAK by DMD. We monitored the convergence of energy distributions for the protein during the simulations to evaluate its equilibration. We repeated each DMD simulation three times to get statically significant data on FAK-LOV2 conformations. We discarded the initial 1 × 10^6^ DMD time steps and considered the last 4 × 10^6^ DMD time steps for the trajectory analysis and movie generation. We rendered all representative images using PyMOL (https://pymol.org/2/)^35^ and Visual Molecular Dynamics^36^. We used a previously identified and validated site for uniRapR insertion^7^. We studied the conformational change of FAK controlled by uniRapR using DMD. We performed DMD simulations of *Ch*-FAK with and without rapamycin. We analyzed the conformations of *Ch*-FAK after 0.3 × 10^6^ time steps.

### Plasmid construction

We amplified the genes encoding uniRapR and LOV2 from Addgene vectors (Addgene plasmids #45381 and #87356, respectively). We used pEGFP-RapR-FAK-YM (Addgene plasmid #25928) and pEGFP-FAK-YM as templates to create the variants. We constructed *Ch*-FAK by replacing the sequences encoding EGFP and RapR in pEGFP-RapR-FAK-YM with sequences encoding mCherry and uniRapR, respectively. We constructed the kinase-dead mutant of *Ch*-FAK by inserting mutagenesis of the plasmid to yield the D546R point mutation. We created *Op*-FAK by inserting the LOV2 sequence into pEGFP-FAK-YM. Two *Op*-FAK variants were created, *Op*-lit-FAK (I510E/I539E mutation in LOV2) and *Op*-dark-FAK (C450A mutation in LOV2). For light experiments, we replaced the sequence encoding EGFP with that encoding mCherry in *Op*-FAK. We created *ChOp*-FAK by inserting the sequence encoding uniRapR into the *Op*-FAK-expressing construct. We used Q5 site-directed mutagenesis kit (NEB) as per manufacturer’s recommendations to create all point mutations. Briefly, plasmid constructs were amplified using the primers containing desired mutations via PCR using Q5 high-fidelity master mix (NEB). PCR product is then treated with KLD enzyme mix (NEB) at room temperature for 5 min. Treated PCR products are then transformed into chemically competent DH5α *Escherichia coli* strain (Zymo Research). Correct clones were confirmed by sequencing (Eton Biosciences). We performed insertions of long sequences encoding LOV2 and uniRapR using the modified megaprimer method described previously^14^. Briefly, gene of interest was PCR amplified in the first step. Resulting PCR products flanked by homologous arms to the targeted cloning site in a plasmid. Using the purified PCR products as mega primer and target cloning vector as template, a second step PCR led to the insertion of gene of interest into the target plasmid site. We further treated the PCR products with KLD1 enzyme mix for 5 min at room temperature and transformed into chemically competent DH5α *E. coli* strain. Cells were grown on LB plates and single clones were enriched and sent for sequencing. Linker sequences were included in primer designs. Supplementary Table 1 lists the primer sequences used in this study. All primers were obtained from Sigma-Aldrich. Supplementary Table 2 shows the uniRapR and LOV2 insertion sites. The region of the *ChOp*-FAK sequence with the inserted LOV2 and uniRapR sequences is shown in Supplementary Table 3.

### Cell culture and transient expression

HeLa (ATCC CCL-2), MDA-MB-231 (ATCC HTB-26), and *FAK*^−/−^ fibroblast cells (ATCC CRL-2644) were maintained in Dulbecco’s modified eagle medium (Lonza) supplemented with 10% fetal calf serum (Gibco) at 37 °C and 5% CO_2_. Cells were passaged when they reach confluency of approximately 90%. For microscopy experiments, 50,000 HeLa, MDA-MB-231, or *FAK*^−/−^ cells were seeded on coverslips coated with 5 µg/mL fibronectin. The following day, cells were transfected with 500 ng of plasmid using JetPrime (Polyplus-transfection) according to the manufacturer’s instructions. We performed the microscopy imaging experiments at 16 h after transfection.

### Elastic micropattern preparation

The protocol used for fibronectin/collagen type-1 micropattern microfabrication has been described^37,38^. Briefly, for elastic hydrogel micropatterning, we initially printed fluorescent dye- and biotin-labeled fibronectin or collagen type-1 on cover glass, then transferred the micropatterns onto hydrogels by crosslinking their biotin tags to streptavidin-conjugated poly(acrylic acid) (PAA)^39^. For high-precision microcontact printing on the intermediate cover glass, we utilized composite elastomeric stamps^40^. We cast a thin layer of non-collapsing hard polydimethylsiloxane (hPDMS; *L* ≤ 0.5 mm) by spin-coating on photo-etched 1 μm master molds to replicate their micropattern bas-reliefs^39^. The hPDMS premix was 3.4 g of VDT-731 (Gelest, Inc.), 18 µL of platinum catalyst (Pt(0)−2,4,6,8-tetramethyl-2,4,6,8-tetravinylcyclotetrasiloxane complex solution, Sigma-Aldrich), and 10 μL of crosslinking modulator (2,4,6,8-tetramethyl-2,4,6,8-tetravinylcyclotetrasiloxane, Sigma-Aldrich). We mixed the freshly prepared hPDMS mixture with 1 g of HMS-301 (Gelest, Inc.) for 30 s on vortex mixer immediately before use. A submicron-thick layer of hPDMS was spin-coated onto the silicone molding matrix, which was then baked for 30 min at 60–70 °C. We poured regular PDMS (1:5 curing agent/base ratio, Sylgard-184, Dow Corning) atop the cured hPDMS as a thick cushioning supporting layer (*h* ~ 7 mm). After curing at 70 °C for 1 h, the layers were peeled from the molding matrix and cut into the 8 × 8 mm^2^ stamps.

Using these composite stamps, we created fibronectin micropatterns on PAA. First, fibronectin was conjugated with (+)-biotin *N*-hydroxysuccinimide ester (Sigma-Aldrich; as specified in the vendor’s protocol) and with a fluorescent tag (Alexa Fluor succinimidyl ester; Invitrogen, Molecular Probes; as per the vendor’s protocol). We then coated microstamps with fibronectin at a concentration of 0.2 mg/mL in phosphate-buffered saline (PBS) by incubation for 40 min at 37 °C in a humid chamber. We gently rinsed stamps in deionized water and dried them under a jet of air or nitrogen immediately before use. Glass-bottomed, 35 mm petri dishes (MatTek Corp.) were activated with 3-(trimethoxysilyl) propyl methacrylate (Sigma-Aldrich) for covalent crosslinking with PAA gels. We added curing catalyst (aminopropyltriethoxysilane (APS)^41^) to 5 μL of PAA premix containing 5% streptavidin–acrylamide (ThermoFisher) of the defined rigidity and sandwiched it between the activated dish and the micropatterned cover glass. In order to finely control PAA mechanical rigidity, we modulated concentrations of 40% acrylamide base (BioRad) and its crosslinking molecular chain 2% bis-acrylamide (BioRad). Additionally, streptavidin–acrylamide (ThermoFisher) was added to a final concentration of 0.133 mg/mL to allow crosslinking of PAA gels with biotinylated proteins of interest. For preparation of 50 µL of PAA gel premixes with storage elastic modulus (*G*′) of 2.3 and 50 kPa, respectively, we mixed the following components: 9.33 or 15 µL 40% acrylamide; 1.88 or 14.40 µL 2% bis-AA; 3.33 or 3.33 µL 2 mg/mL streptavidin-acrylamide; 5 or 5 µL 10× PBS; 30 or 11.17 µL deionized milli-Q water; 0.1 or 0.1 µL tetramethylethylenediamine; and 1 or 1 µL 10% APS. We incubated the cured PAA sandwiches in room temperature deionized water for 1 h to release the hypotonic cover glass from the PAA gel. After cover glass removal, dish-bound gels retained fluorescent fibronectin or collagen type-1 micropatterns. We added the transfected cells to the micropatterns and waited for 2–3 h before performing the imaging experiments.

### Inhibition studies

Stock solutions of (-)-blebbistatin (Sigma-Aldrich) and FAK inhibitor 14 (Tocris) were prepared 50 mM in DMSO. Before addition to cells, we incubated the solutions for 20 min in a 37 °C water bath to dissolve the drug completely, and then filtered the solutions. Controls were incubated with corresponding amounts of DMSO. After the treatment, we fixed the cells with cold DMEM with 4% PFA. We stained F-actin with Alexa Fluor phalloidin conjugates (ThermoFisher Scientific; 10 U/mL in 1% BSA PBS) and chromatin with 1:1000 Hoechst solution (Tocris).

### Imaging, data processing, and analysis

We performed high-resolution 3D and 2D imaging for cell morphometric analysis on a Leica SP8 confocal scan head with 40X immersion oil objective with sequential excitation by 405, 488, 561, and 647 nm lasers. We collected the fluorescent signal through a 1.2 AU pinhole using adjustable emission filters with automatic pixel size adjustment with Leica Microsystems software. We used the 561 nm filter to image the mCherry constructs and the 488 nm filter to image the EGFP constructs. For blue-light irradiation experiments, we used the mCherry channel for focusing and 488 nm laser beam (2% laser intensity). We performed depth analysis and morpho-mechanical and morphometric analyses automatically and/or manually utilizing Leica Microsystems software (Leica Application Suite X v5.0.2) and ImageJ/FiJi open-source software. Video sequences were analyzed with the ImageJ stacks plug-in. We analyzed focal adhesions using the plug-in “particle analyzer”^42^. Fold changes were calculated from the total numbers of focal adhesions and average sizes of the focal adhesions in individual cells. For rapamycin experiments, we calculated fold change by normalizing the post-treatment values to pre-treatment values. For irradiation experiments, we calculated fold changes by normalizing the post-irradiation values to pre-irradiation values. We analyzed 10–15 cells for each experiment. We conducted all live-cell imaging on-stage in a Tokai Hit STGX-GSI2-SET active CO_2_ Z-galvo incubator.

### Western Blot

We treated cells expressing *Ch*-FAK constructs with 50 nM rapamycin or solvent control (ethanol). We exposed cells expressing *Op*-FAK or control constructs to blue light for 60 min. We lysed the cells using RIPA buffer (ThermoFisher Scientific), in the presence of protease and phosphatase inhibitor cocktail set (Thermo scientific). We measured the Protein concentration with BCA Assay Kit (Thermo scientific) following manufacturer’s instructions. We run the proteins on home-made SDS-Page gels. We transferred gels on PVDF membrane paper using 1x transfer buffer (Bio-Rad). We blocked the membrane with 5% BSA for 1 h prior to addition of primary antibody. We used the following primary antibodies according to the manufacturer’s instructions: Phospho-FAK (Y397) (Sigma-Aldrich, #ABT135), Phospho-paxillin (Y31) (Invitrogen, #44-720 G), FAK (Santa Cruz Biotechnology, #SC557), Paxillin (Sigma-Aldrich, #SAB4502553), β-actin (Cell Signaling, #8457). Horseradish peroxidase-linked secondary antibodies: Goat Anti-Mouse IgG Antibody, Peroxidase Conjugated (Sigma-Aldrich, #AP124P) and Goat anti-Rabbit IgG (H + L) Secondary Antibody, HRP conjugated (ThermoFisher Scientific, #31460). We used all the primary antibodies in a dilution of 1:1000. Anti-β-actin used in 1:5000. We used all the secondary antibodies in 1:2500 dilution. We performed chemiluminescence detection by using the Pierce ECL Western Blotting Substrate (Thermo Scientific). We collected the fluorescence signals using Image Bio-Rad ChemiDoc imaging system. The uncropped full length scans for all western blot are provided in the Source Data file.

### Migration analysis

We performed cell tracking on PAA (*G*′ of 8.6 kPa) surface-imprinted with fluorescently labeled collagen type-1 micropatterns using MDA-MB-231 cells that express *Ch*-FAK. We performed live-cell imaging using a Tokai Hit STGX-GSI2-SET active CO_2_ Z-galvo incubator. The 488 nm filter was used to image EGFP, and the 561 nm filter was used to image mCherry using a 10x objective. We maintained the cells at 37 °C in 5% CO_2_ for the duration of live-cell imaging (8–10 h). We analyzed the results using TrackMate plug-in in ImageJ by tracking single cells manually.

### Statistics and reproducibility

We performed all statistical analyses by unpaired two-tailed Student’s *t*-test using GraphPad Prism 8 v8.2.1 software and Microsoft Excel v16.49. The levels of significance are denoted as ^*^P ≤ 0.05, ^**^P ≤ 0.01, ^***^P ≤ 0.001, ^****^P ≤ 0.0001, and NS not significant (*P* > 0.05). We used GraphPad Prism 8 version 8.2.1 and Adobe Illustrator to draw and assemble the figures. All experiments showing representative microscopy data (Figs. 2b, d, 3a, 4a–c, and 6b, d, e and Supplementary Figs. 3, 4, 6–11a, b, and 12a, b) and immunoblots (Supplementary Figs. 5 and 14) were repeated at least three times with similar results. Independent replicates refer to independent cell samples seeded, transfected, treated, and analyzed on different days.

### Reporting summary

Further information on research design is available in the Nature Research Reporting Summary linked to this article.

## Supplementary information

Supplementary information.

Description of Additional Supplementary Files.

Supplementary Video 1.

Supplementary Video 2.

Supplementary Video 3.

Reporting summary.

## Data Availability

We used RCSB PDB protein data bank to obtain 2J0J and 2V0U PDB structures (https://www.rcsb.org/structure/2J0J and https://www.rcsb.org/structure/2V0U). Plasmids harboring important genes used in this study are available from Addgene: uniRapR, FAK, and LOV2 from Addgene plasmids #45381, #25928, and #87356, respectively. The data that support the findings of this study are in Supplemental data. All other data are available from the corresponding author on reasonable request. Source data are provided with this paper.

## Source data

Source Data.
