# Supplementary material for: Two-input protein logic gate for computation in living cells
Source: Nat Commun. 2021 Nov 16;12:6615. doi: 10.1038/s41467-021-26937-x (PMC8595391; doi:10.1038/s41467-021-26937-x)
Supplement: Supplementary file 1 — Supplementary information. [file 41467_2021_26937_MOESM1_ESM.pdf]

## Supplementary Material

**Supplementary Table 1. List of primers used in the study.**

| SN | Primer Name | Sequence (5' to 3') |
|---|---|---|
| 1 | UNIRAPR-FAK-F | cagtttggagatgtacatcaaggcgtgtacggcccaggaacctgcgtggtgcactacacc |
| 2 | UNIRAPR-FAK-R | gtttttacatgttttgattgcaacagccaaacctgggccttccagttttagaagctccac |
| 3 | LOV2-G-FAK-F | aaagagtgcttcaagtgtgcccttgggtcaggcttggctactacacttgaacgt |
| 4 | LOV2-G-FAK-R | gattgccaattccacagaaatgatccaactgccaagttcttttgccgcctcatc |
| 5 | LOV2-GP-FAK-F | aaagagtgcttcaagtgtgcccttgggtcaggcccattggctactacacttgaacgt |
| 6 | LOV2-GP-FAK-R | gattgccaattccacagaaatgatccaacttgggccaagttcttttgccgcctcatc |
| 7 | LOV2-GSG-FAK-F | aaagagtgcttcaagtgtgcccttgggtcaggctcaggcttggctactacacttgaacgt |
| 8 | LOV2-GSG-FAK-R | gattgccaattccacagaaatgatccaacttcctgagccaagttcttttgccgcctcatc |
| 9 | LOV2-NL-FAK-F | aaagagtgcttcaagtgtgcccttgggtcattggctactacacttgaacgt |
| 10 | LOV2-NL-FAK-R | gattgccaattccacagaaatgatccaactaagttcttttgccgcctcatc |
| 11 | LOV2-GPGSG-FAK-F | aaagagtgcttcaagtgtgcccttgggtcaggcccaggatccggattggctactacacttgaa |
| 12 | LOV2-GPGSG-FAK-R | gattgccaattccacagaaatgatccaactgccagaacctgggccaagttcttttgccgcctcatc |
| 13 | LOV2-GPGPG-FAK-F | gatgaggcggcaaaagaacttggcccaggtccaggcagttggatcatttctgtggaattggcaatc |
| 14 | LOV2-GPGPG-FAK-R | attgccaattccacagaaatgatccaactgcctggacctgggccaagttcttttgccgcctcatc |
| 15 | LOV2-GPG-FAK-F | aaagagtgcttcaagtgtgcccttgggtcaggcccaggattggctactacacttgaacgt |
| 16 | LOV2-GPG-FAK-R | gattgccaattccacagaaatgatccaactacctgggccaagttcttttgccgcctcatc |
| 17 | FAK_WT_YM-F | gaggccaggggtaatgccctagag |
| 18 | FAK_WT_YM-R | ccaggcggatcgcctaatttccaaac |
| 19 | D546R_KD_F | tgttcacaggagaattgctgctc |
| 20 | D546R_KD_R | aatcttttgctctccagatac |
| 21 | LOV_LIT_I510E-F | ccagtactttgaaggggttcagttg |
| 22 | LOV_LIT_I510E-R | ccagtactttgaaggggttcagttg |
| 23 | LOV_LIT_I539E-F | tgcagaaaatgaagatgaggcgg |
| 24 | LOV_LIT_I539E-R | gttttcttaatcagcatgac |
| 25 | LOV_DARK_C450A-F | gggaagaaacgccaggtttctacaag |
| 26 | LOV_DARK_C450A-R | aaaatttcttcacggctatattc |
| 27 | CHERRY-F | gatccgctagcgctaccggtcgccaccatggtgagcaagggcgaggag |
| 28 | CHERRY-R | agctgccattccagatctgagtccggacttgtacagctcgtccatgcc |
| 29 | LOV2-LOOP3-FAK-F | aaaggaatgctacaactcaaaatcgctggtttggctactacacttgaacgtatt |
| 30 | LOV2-LOOP3-FAK-R | tggtgccgtcacggtcagaggctcaggagcaagttcttttgccgcctcatc |
| 31 | FAK-ULOV2-GPG-F | cagtttggagatgtacatcaaggcgtgtacggcccaggattggctactacacttgaacgtatt |
| 32 | FAK-ULOV2-GPG-R | gtttttacatgttttgattgcaacagccaaacctgggccaagttcttttgccgcctcatc |
| 33 | FAK-ULOV2-F | cagtttggagatgtacatcaaggcgtgtacttggctactacacttgaacgtatt |
| 34 | FAK-ULOV2-R | gtttttacatgttttgattgcaacagccaaaagttcttttgccgcctcatc |

**Supplementary Table 2. Insertion sites, linkers and loops used for the study.**

| Target domain | Domain inserted | Inserted linker | Insertion loop | Insertion site |
|---|---|---|---|---|
| Kinase | uniRapR | GPG | Loop 1 | FGDVHQGVY*LAVAIKTC |
| FERM | LOV2 | GSG | Loop 2 | KECFKCALGS*SWIISVE |
| FERM | LOV2 | - | Loop 2 | KECFKCALGS*SWIISVE |
| FERM | LOV2 | GPGSG | Loop 2 | KECFKCALGS*SWIISVE |
| FERM | LOV2 | G | Loop 2 | KECFKCALGS*SWIISVE |
| FERM | LOV2 | GPG | Loop 2 | KECFKCALGS*SWIISVE |
| FERM | LOV2 | GP | Loop 2 | KECFKCALGS*SWIISVE |
| FERM | LOV2 | GPGPG | Loop 2 | KECFKCALGS*SWIISVE |
| FERM | LOV2 | - | Loop 3 | GMLQLKIAG*APEPLTVT |

* Insertion site

**Supplementary Table 3. Partial sequence of *ChOp*-FAK showing the inserted uniRapR and LOV2 sequences.**

| |
|---|
| NFFYQQVKSDYMQEIADQVDQEIALKLGCLEIRRSYWEMRGNALEKKSNYEVLEKDVGLKRFFPKSLLDSVKAK<br>TLRKLIQQTFRQFANLNREESILKFFEILSPVYRFDKECFKCALGSGLATTLERIEKNFVITDPRLPDNPIIFASDSFLQ<br>LTEYSREEILGRNCRFLQGPETDRATVRKIRDAIDNQTEVTVQLINYTKSGKKFWNLFHLQPMRDQKGDVQYFI<br>GVQLDGTEHVRDAAEREGVMLIKKTAENIDEAAKELGSWIISVELAIGPEEGISYLTDKGCNPTHLADFNQVQTI<br>QYSNSEDKDRKGMLQLKIAGAPEPLTVTAPSLTIAENMADLIDGYCRLVNGATQSFIIRPQKEGERALPSIPKLAN<br>SEKQGMRTHAVSVSETDDYAEIIDEEDTYTMPSTRDYEIQRERIELGRCIGEGQFGDVHQGVYGPGTCVVHYTG<br>MLEDGKKFDSSRDRNKPFKFMLGKQEVIRGWEEGVAQMSVGQRAKLTISPDYAYGATGHGSGSGSGVKDLLQ<br>AWDLYYHVFRRISGPPGPGSGLWHEMWHEGLEEASRLYFGERNVKGMFEVLEPLHAMMERGPQTLKETSFN<br>QAYGRDLMEAQEWCRKYMKSGSSGGSGSGIIPPHATLVFDVELLKLEGPGLAVAIKTCKNCTSDSVREKFLQEA<br>LTMRQFDHPHIVKLIGVITENPVWIIMELCTLGELRSFLQVRKYSLDLASLILYAYQLSTALAYLESKRFVHRDIAAR<br>NVL |
| Black- FAK; Blue- LOV2; Red- UniRapR; Purple-linkers |

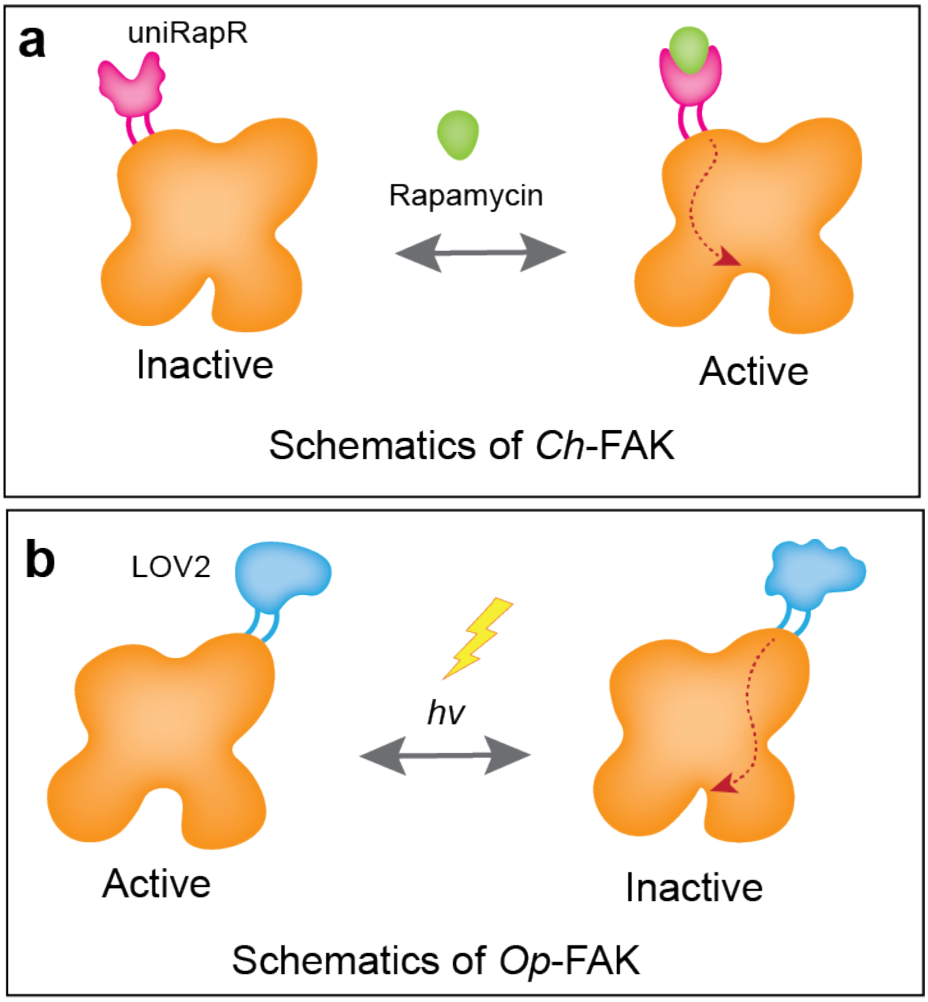


**Supplementary Fig. 1**. **Rapamycin and light mediated control of FAK activity. a,** uniRapar domain is allosterically inserted into the kinase domain in *Ch*-FAK. Addition of rapamycin reduces the distortion introduced by the uniRapR domain, leading to activation of FAK. **b**, LOV2 domain is allosterically inserted into the FERM domain in *Op*-FAK. Light induces confirmational change in LOV2, leading to distortion of the FAK and resulting in its inactivation.

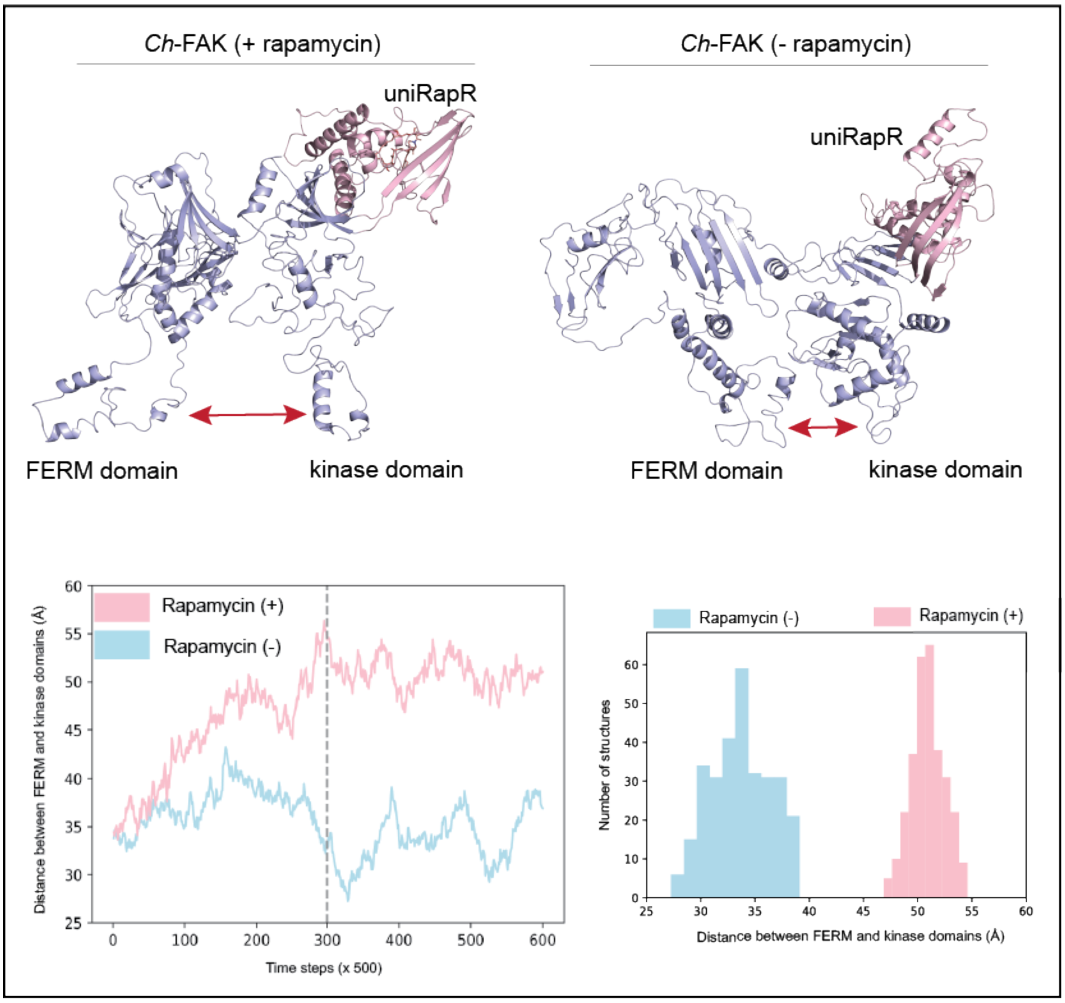


**Supplementary Fig. 2. DMD Simulations of *Ch*-FAK in presence and absence of rapamycin**. Top, snapshots from the DMD simulations showing conformations of *Ch*-FAK, in presence (left) and absence (right) of rapamycin. The distance between the two domains in presence of rapamycin is indicative of activation of the protein. Bottom, the distances between FERM and kinase domains in *Ch*-FAK (in presence and absence of rapamycin) quantified during DMD simulations. Source data are provided as a Source Data file.

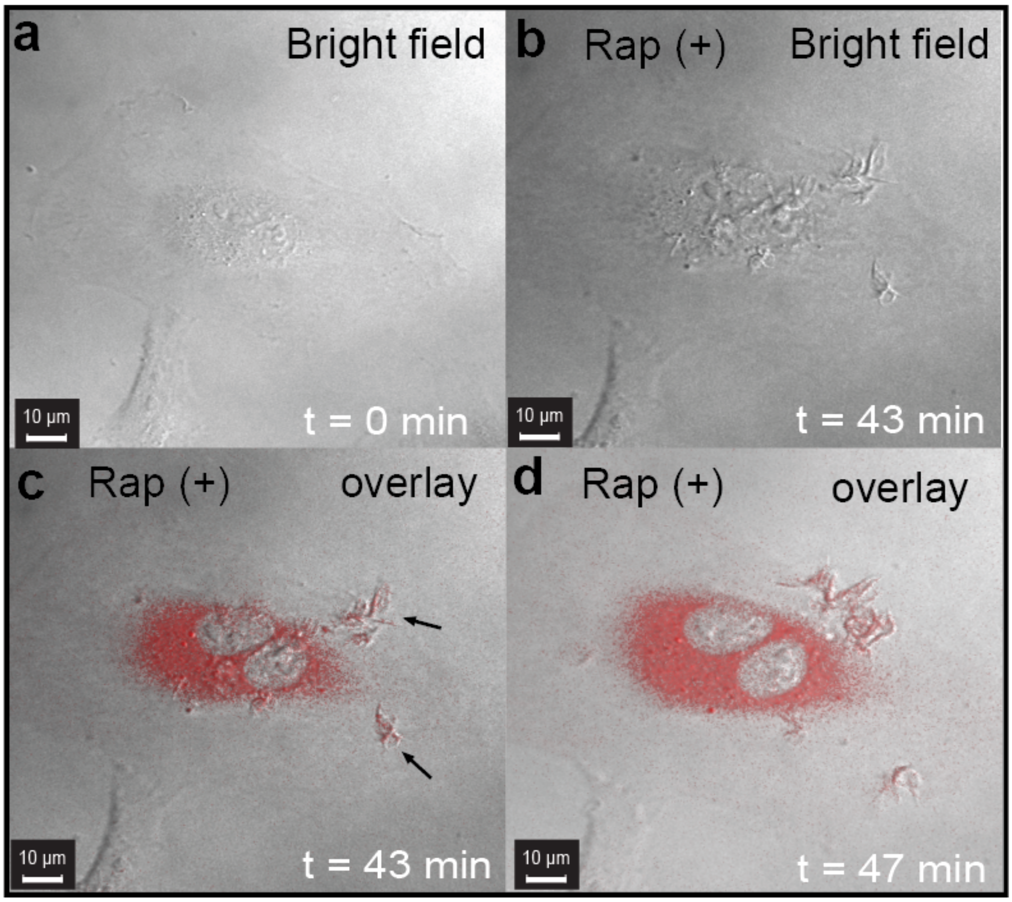


**Supplementary Fig. 3. Localization of activated *Ch*-FAK in the dorsal ruffles**. **a**-**b** Time-lapse bright field imaging data for the HeLa cells that express *Ch*-FAK before and after treatment with 50 nM rapamycin. Rapamycin was added at 30$^{th}$ min during the live-cell imaging. Ruffles are indicated by arrows. **c-d,** Overlay of bright field and fluorescent channels at two different time points after rapamycin activation. Arrows indicate the localization of mCherry tagged *Ch*-FAK in the ruffles. Scale bar, 10 µm.

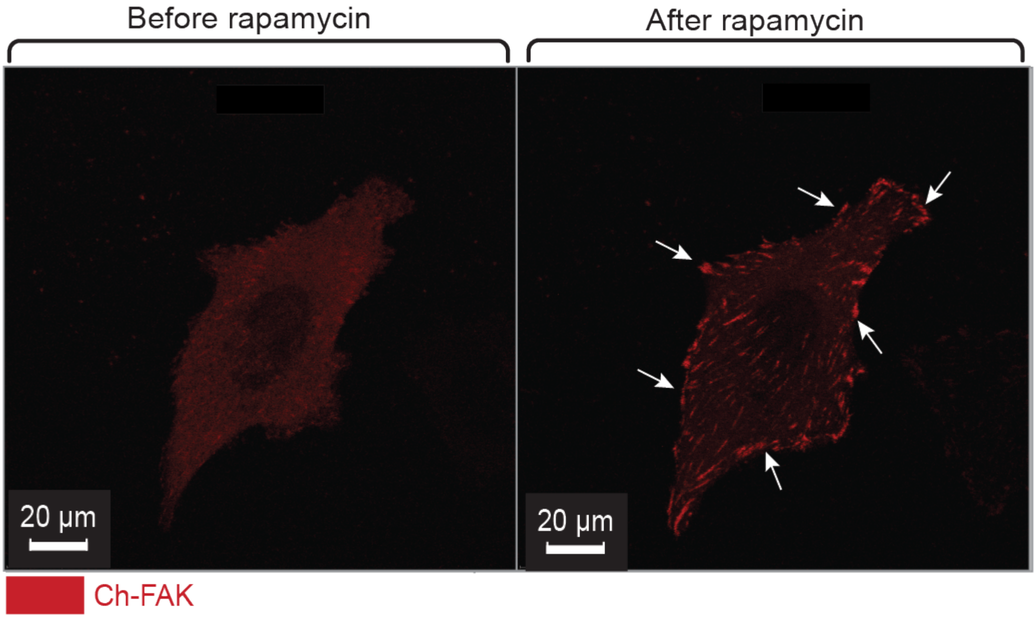


**Supplementary Fig. 4. Rapamycin induced activation of *Ch*-FAK**. Images of *FAK*$^{-/-}$ fibroblasts that express *Ch*-FAK before and after 50 nM rapamycin treatment. Activated *Ch*-FAK translocates to focal adhesions to form enlarged, late focal adhesions, which are indicated by arrows. Scale bar, 20 µm.

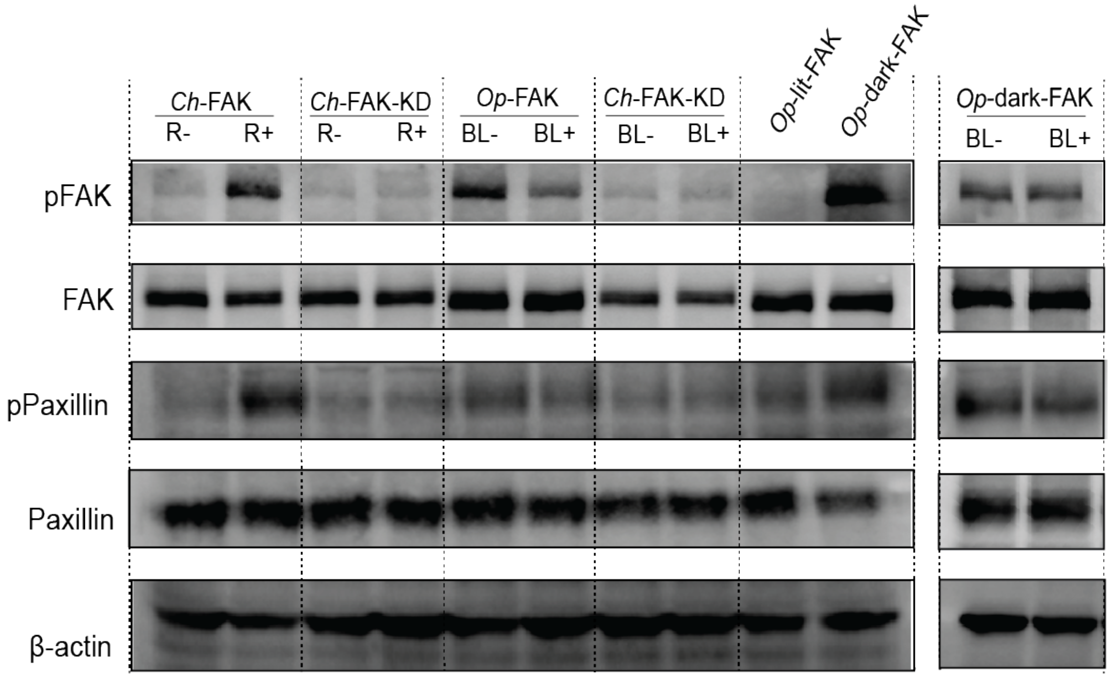


**Supplementary Fig. 5. Immunoblot analysis of *Ch*-FAK and *Op*-FAK constructs.** HeLa cells expressing *Ch*-FAK constructs were treated with 50 nM rapamycin or ethanol for 1 hour. HeLa cells expressing *Op*-FAK and control constructs were exposed to blue light for 1 hour. Phosphorylation levels of *Ch*-FAK (Y397), *Op*-FAK (Y397), *Op*-dark-FAK (Y397), *Op*-lit-FAK (Y397) and Paxillin (Y31) were tested using cell lysates. R, rapamycin; BL, blue light. Source data are provided as a Source Data file.

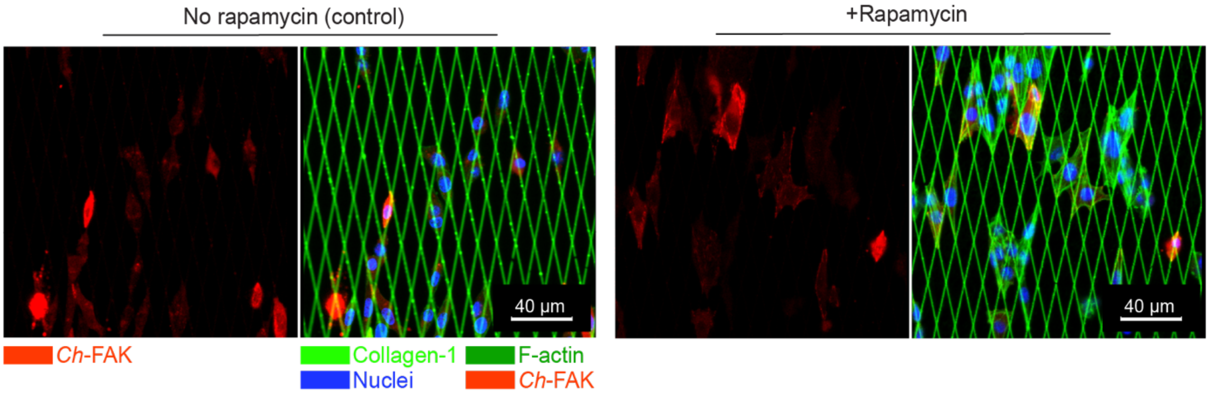


**Supplementary Fig. 6. Cell morphological response to FAK activation on the structurally standardized biomimetic collagen type-1 fibers**. Fluorescent micrographs for *Ch*-FAK-expressing *FAK*$^{-/-}$ cells with (right) and without (left) rapamycin treatment on 2D collagen type-1 micropatterns. Scale bar, 40 µm.

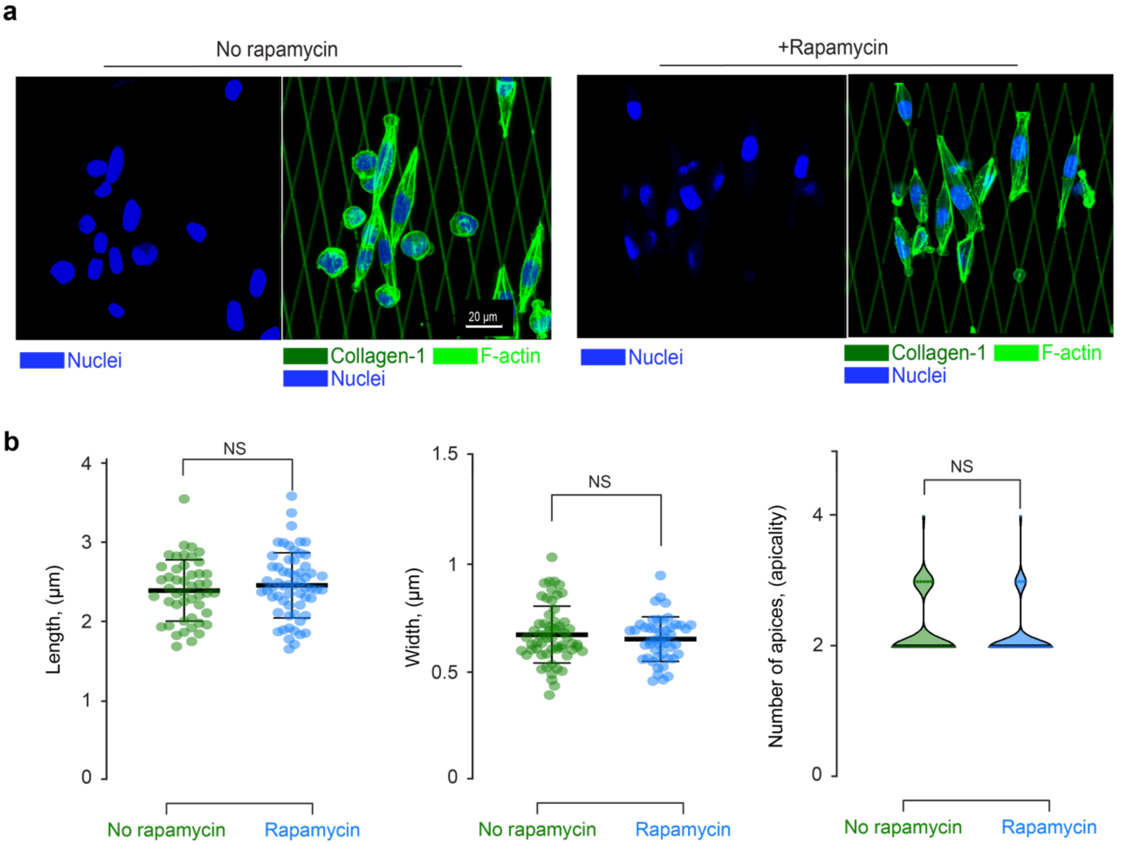


**Supplementary Fig. 7. Cell morphological response to rapamycin treatment on the structurally standardized biomimetic collagen type-1 fibers. a,** Representative cell fluorescent micrographs for non-transfected *FAK*$^{-/-}$ cells with (right) and without (left) rapamycin treatment on 2D collagen type-1 micropatterns. Scale bar, 20 µm. **b,** Length, width, and apicality of non-transfected *FAK*$^{-/-}$ fibroblasts with and without rapamycin treatment. n=47 cells for No Rapamycin and n=50 cells for rapamycin treated conditions from 3 independent experiments. P=0.3942 for length, P=0.624 for width, and P=0.311 for apicality calculated by unpaired two-tailed Student's t-test. NS, non significant. Source data are provided as a Source Data file.

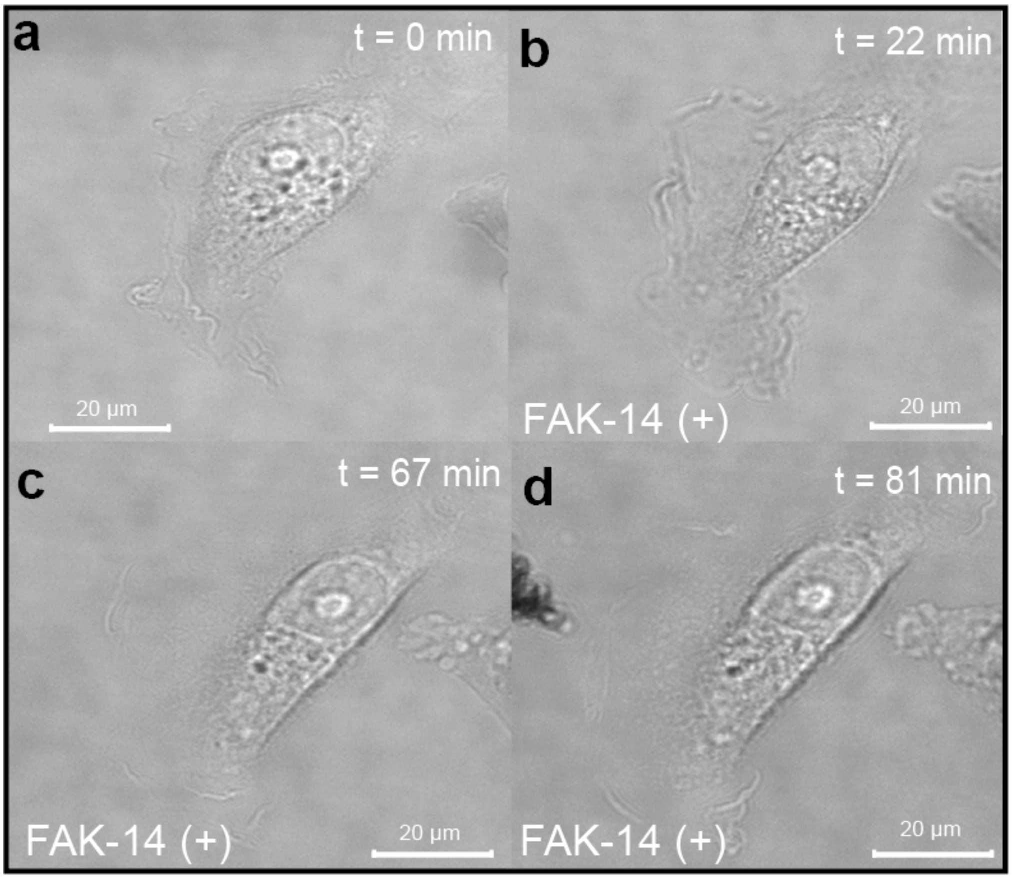


**Supplementary Fig. 8**. **Time-lapse bright field imaging data for the effect of FAK inhibitor 14 treatment on non-transfected *FAK*$^{-/-}$ cells. a,** Image of non-transfected *FAK*$^{-/-}$ fibroblast before FAK inhibitor 14 treatment. **b-d**, Images of non-transfected *FAK*$^{-/-}$ fibroblast after the FAK inhibitor 14 treatment. FAK inhibitor 14 added at 15$^{th}$ minute. Scale bar, 20 µm.

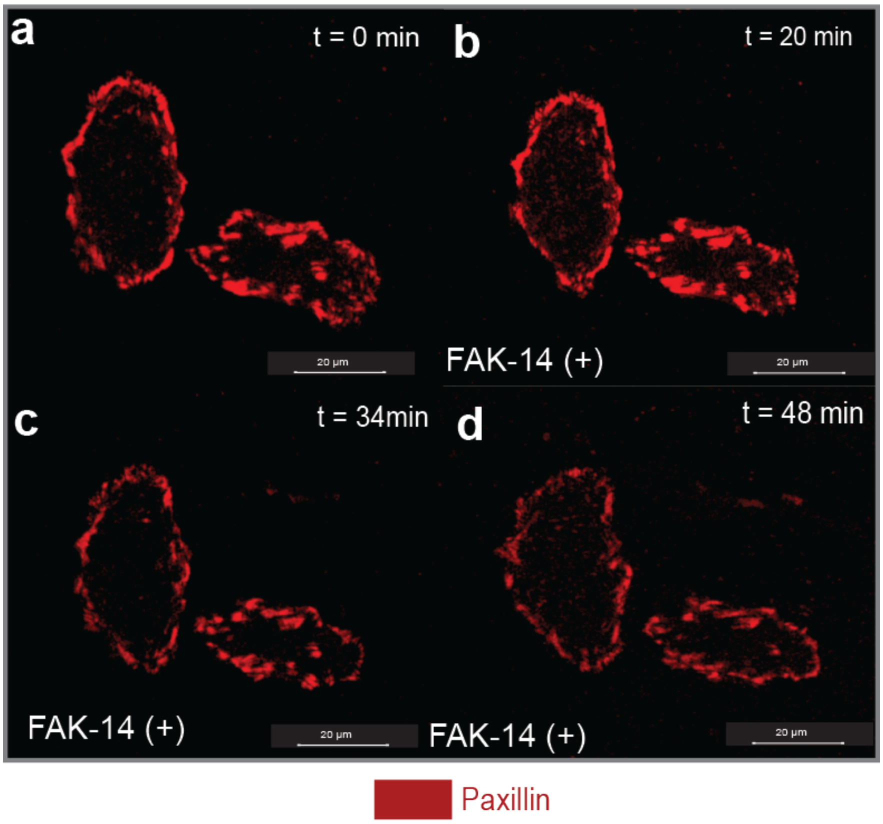


**Supplementary Fig. 9**. **Time-lapse fluorescent imaging data for the effect of FAK inhibitor 14 treatment on mCherry-Paxillin transfected *FAK$^{-/-}$* cells. a,** Image of Paxillin expressing *FAK$^{-/-}$* fibroblast before FAK inhibitor 14 treatment. **b-d**, Images of Paxillin expressing *FAK$^{-/-}$* fibroblast after the FAK inhibitor 14 treatment. FAK inhibitor 14 added at 15$^{th}$ minute. Scale bar, 20 µm.

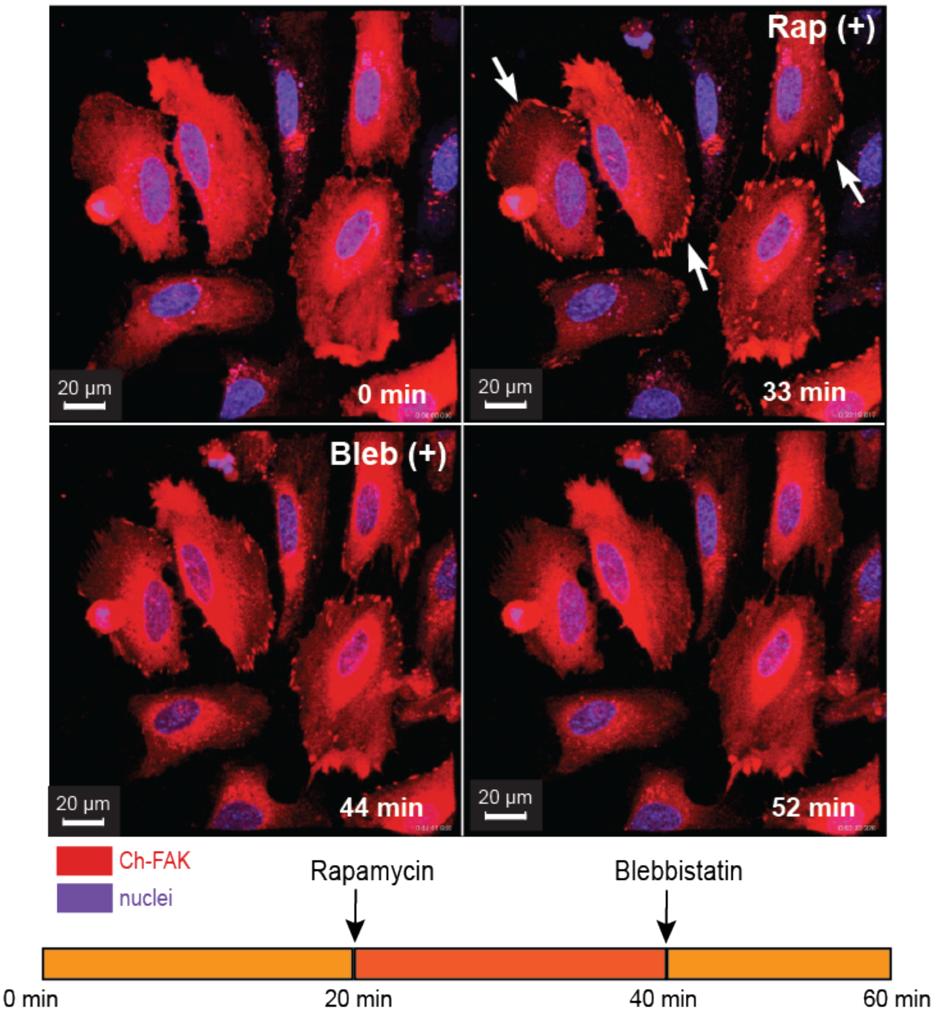


**Supplementary Fig. 10. Time-lapse fluorescent imaging data for the effect of blebbistatin treatment on *Ch*-FAK activated cells.** A 60 min time-lapse fluorescent imaging data showing the effect of blebbistatin treatment on rapamycin treated *Ch*-FAK expressing HeLa cells. Rapamycin was added at 20$^{th}$ min and blebbistatin was added at 40$^{th}$ min. Addition of 50 nM rapamycin to *Ch*-FAK-expressing HeLa cells activated *Ch*-FAK resulting in formation of enlarged, late focal adhesions (33 min image, focal adhesions are indicated by arrows). Addition of blebbistatin to *Ch*-FAK activated cells resulted in degradation of focal adhesions (44 and 52 min images). Scale bar, 20 µm.

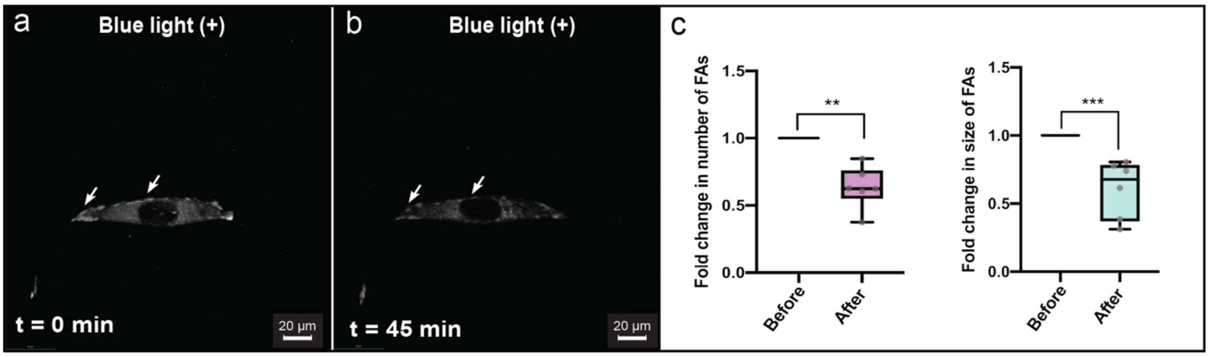


**Supplementary Fig. 11**. **Light induced Inactivation of *Op*-FAK on a soft (fibronectin coated 2.3 kPa PAA hydrogel) surface.**

**a-b,** Time-lapse fluorescent imaging data for the blue light (488 nm) induced inactivation *Op*-FAK in *FAK*$^{-/-}$ cells on a fibronectin coated 2.3 kPa PAA hydrogel surface. Arrows indicate the focal adhesions at 0$^{th}$ min and 45$^{th}$ min. Scale bar, 40 µm. **c,** Normalized quantification of focal adhesions in *Op*-FAK transfected *FAK*$^{-/-}$ cells in response to blue light. FAs indicates focal adhesions. Data represent box plots and individual data points. Data represent box plots and individual data points. Box plots show the median (center line), first and third quartiles (box edges), while the whiskers going from each quartile to the minimum or maximum. n=6 cells for total FAs and average size of FAs from 3 independent experiments; ***P ≤ 0.001, and **P ≤ 0.01 by unpaired two-tailed Student's t-test. Source data are provided as a Source Data file.

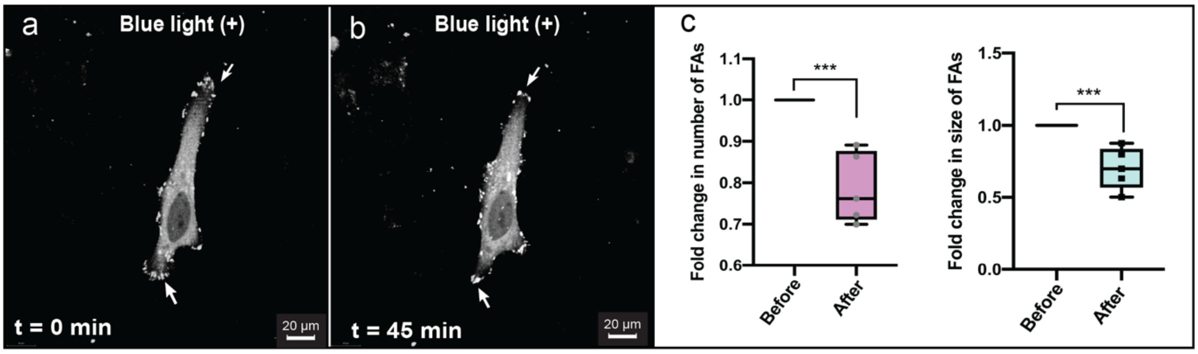


**Supplementary Fig. 12**. **Light induced Inactivation of *Op*-FAK on a collagen type-I coated glass surface.**

**a-b,** Time-lapse fluorescent imaging data for the blue light (488 nm) induced inactivation *Op*-FAK in *FAK*$^{-/-}$ cells on a collagen-type 1 coated glass surface. Arrows indicate the focal adhesions at 0$^{th}$ min and 45$^{th}$ min. Scale bar, 40 µm. **c,** Normalized quantification of focal adhesions in *Op*-FAK transfected *FAK*$^{-/-}$ cells in response to blue light. FAs indicates focal adhesions. Data represent box plots and individual data points. Box plots show the median (center line), first and third quartiles (box edges), while the whiskers going from each quartile to the minimum or maximum. n=5 cells for total FAs and average size of FAs from 3 independent experiments; ***P ≤ 0.001 by unpaired two-tailed Student's t-test. Source data are provided as a Source Data file.

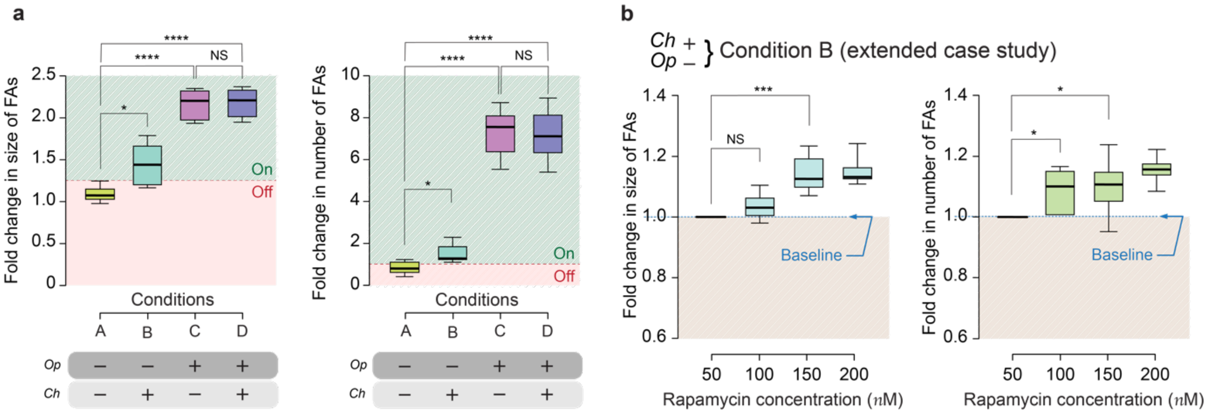


**Supplementary Fig. 13**. **Testing the functionality of *ChOp*-FAK**. **a,** Quantification of average size and total number of focal adhesions in *ChOp*-FAK-expressing $FAK^{-/-}$ cells under conditions A, B, C, and D. The pink box indicates inactivation and green box indicates activation of FAK. Data were normalized to condition A. Data represent box plots. Box plots show the median (center line), first and third quartiles (box edges), while the whiskers going from each quartile to the minimum or maximum. n=8 cells for total FAs and average size of FAs from 3 independent experiments. For total number of FAs, *P=0.0388 for A and B, ****P=5.6 x $10^{-11}$ for A and C, ****P=2.7 x $10^{-10}$ for A and D, P=0.932 for C and D. For Average size of FAs, *P=0.0019 for A and B, ****P=9.7 x $10^{-10}$ for A and C, ****P=2.1 x $10^{-9}$ for A and D, P=0.854 for C and D conditions calculated by unpaired two-tailed Student's t-test. NS, not significant. **b**, Quantification of the average size and total number of focal adhesions in $FAK^{-/-}$ cells that express *ChOp*-lit-FAK in response to increasing concentration of rapamycin. Data represent box plots. Box plots show the median (center line), first and third quartiles (box edges), while the whiskers going from each quartile to the minimum or maximum. n=6 cells for total FAs and average size of FAs from 3 independent experiments. For total number of FAs, *P=0.022 for 50 and 100, *P=0.024 for 50 and 150. For Average size of FAs, P=0.085 for 50 and 100, ***P=0.025 for 50 and 150 calculated by unpaired two-tailed Student's t-test. FAs, Focal adhesions. Source data are provided as a Source Data file.

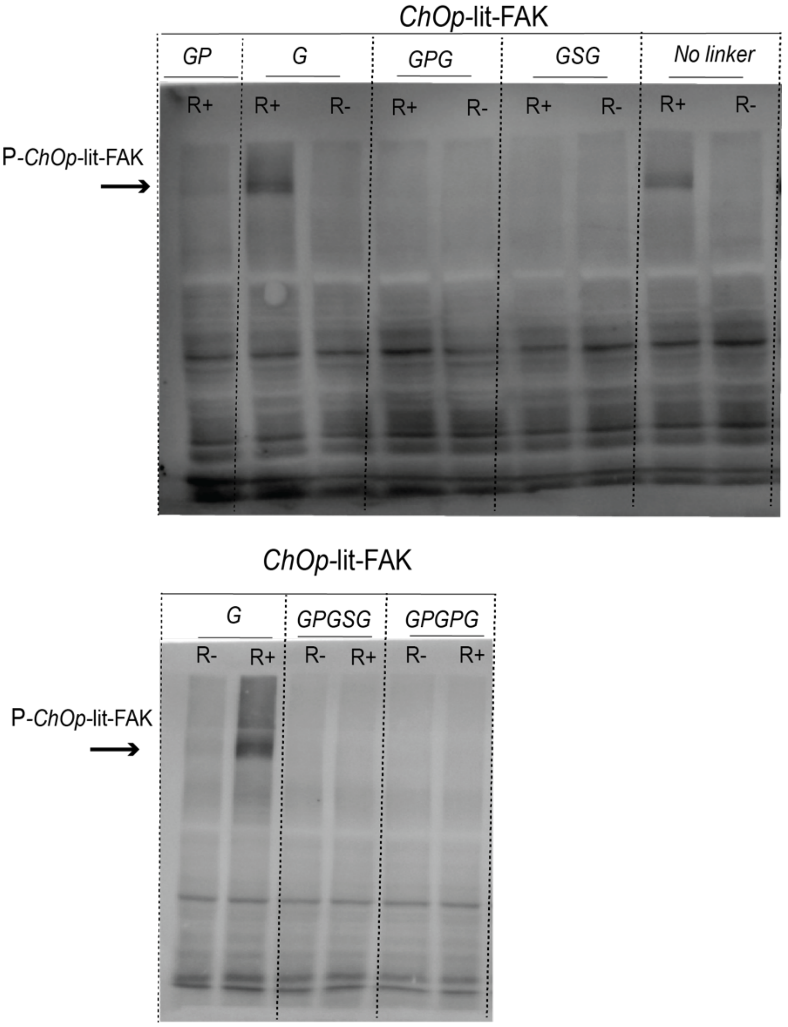


**Supplementary Fig. 14**. **Western blot screening of linker optimized *ChOp*-FAK.**

Optimization of *ChOp*-FAK was performed by inserting various linkers at the N and C-terminus of LOV2 and screening the phosphorylation level (Y397) of *ChOP*-lit-FAK in presence and absence of rapamycin in *FAK*$^{-/-}$ cells. G, P, and S denote glycine, proline, and serine, respectively. P-*ChOp*-lit-FAK indicates Y397-phosphorylated *ChOp*-lit-FAK. The arrow indicates phosphorylated FAK. R- denotes the absence of rapamycin and R+ denotes the presence of 50 nM rapamycin. ‘GP’ linker was used in the initial design. Source data are provided as a Source Data file.

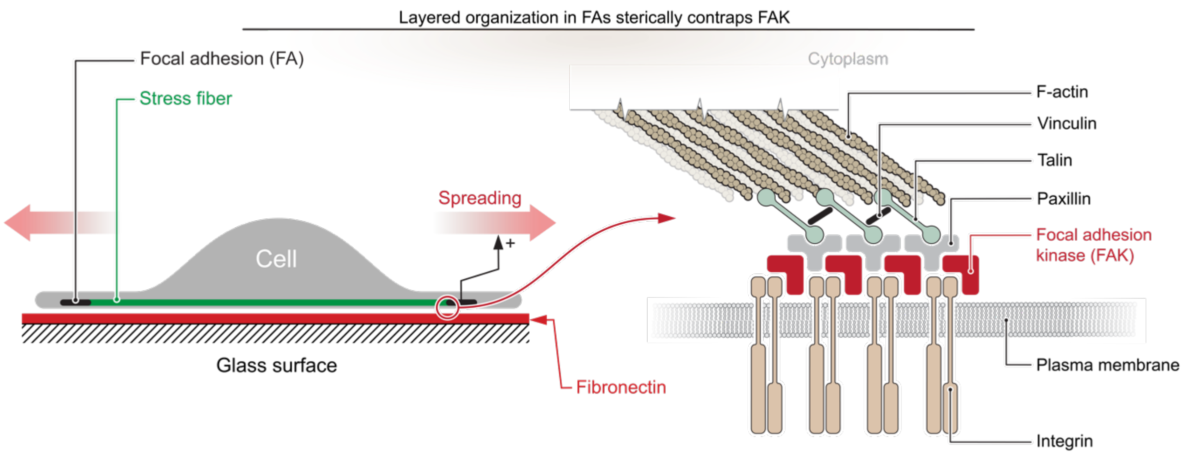


**Supplementary Fig. 15**. **Schematic of focal adhesion organization in a cell**. Focal adhesions are large, densely packed complexes located in cells next to the surface. In focal adhesions, the transmembrane protein integrin binds to the ECM outside the cell and to the stress fibers through talins inside the cell. Upon activation, FAK molecules translocate from cytoplasm to the focal adhesions, where it interacts with several other proteins near the surface to which the cell is adhered.

Uncropped Immunoblot scans

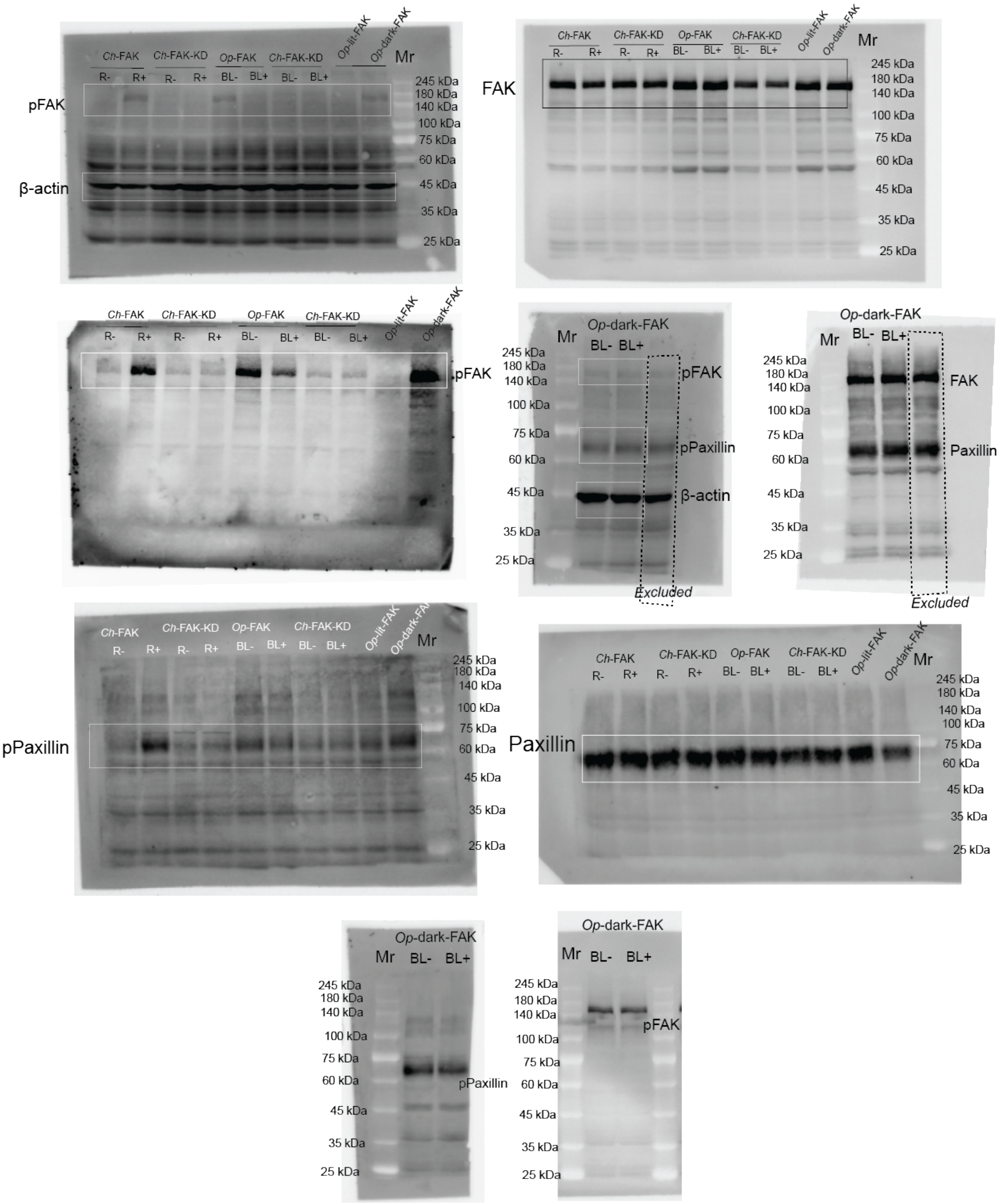


**Uncropped scans for Supplementary Fig. 5. Immunoblot analysis of *Ch*-FAK and *Op*-FAK constructs.** Source data are provided as a Source Data file.

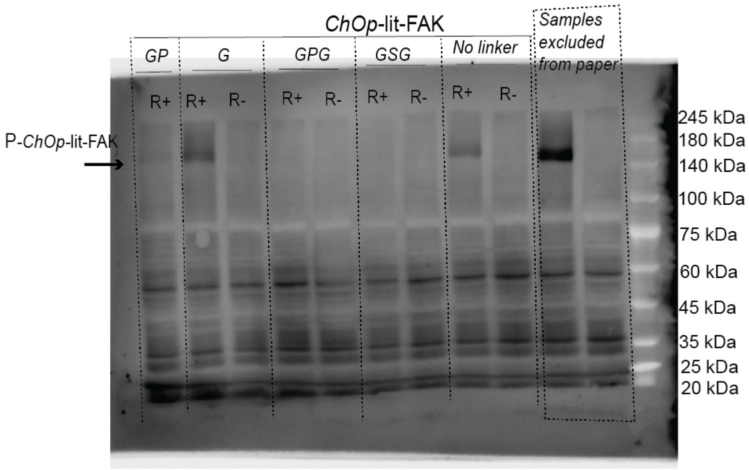


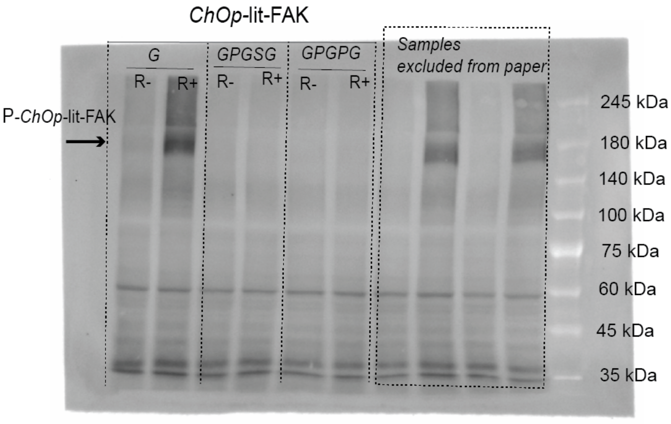


Anti-phospho-FAK (Tyr 397) antibody, (Millipore Sigma #ABT135)

**Uncropped scans for Supplementary Fig. 14**. **Western blot screening of linker optimized *ChOp*-FAK.** Source data are provided as a Source Data file.
