# Supplementary material for: Two-input protein logic gate for computation in living cells
Source: Nat Commun. 2021 Nov 16;12:6615. doi: 10.1038/s41467-021-26937-x (PMC8595391; doi:10.1038/s41467-021-26937-x)
Supplement: Supplementary file 2 — Description of Additional Supplementary Files. [file 41467_2021_26937_MOESM2_ESM.pdf]

Title: Supplementary Movie 1.
Description: Rapamycin induced activation of *Ch*-FAK in HeLa cell. *Ch*-FAK was translocated to focal adhesions upon rapamycin activation resulting in formation of enlarged, late focal adhesions. Cells were visualized using mCherry tagged *Ch*-FAK.

Title: Supplementary Movie 2.
Description: Migration of rapamycin untreated *Ch*-FAK-transfected MDA-MB-231 cells (left panel) along the collagen type-1 micropatterns (right panel).

Title: Supplementary Movie 3.
Description: Migration of rapamycin treated *Ch*-FAK-transfected MDA-MB-231 cells (left panel) along the collagen type-1 micropatterns (right panel).
