# Supplementary material for: Two-input protein logic gate for computation in living cells
Source: Nat Commun. 2021 Nov 16;12:6615. doi: 10.1038/s41467-021-26937-x (PMC8595391; doi:10.1038/s41467-021-26937-x)
Supplement: Supplementary file 6 — Reporting summary. [file 41467_2021_26937_MOESM6_ESM.pdf]

nature portfolio

Corresponding author(s): Nikolay Dokholyan

Last updated by author(s): Oct 24, 2021

Reporting Summary

Nature Portfolio wishes to improve the reproducibility of the work that we publish. This form provides structure for consistency and transparency in reporting. For further information on Nature Portfolio policies, see our Editorial Policies and the Editorial Policy Checklist.

Statistics

For all statistical analyses, confirm that the following items are present in the figure legend, table legend, main text, or Methods section.

n/a	Confirmed	
☐	☒	The exact sample size (n) for each experimental group/condition, given as a discrete number and unit of measurement
☐	☒	A statement on whether measurements were taken from distinct samples or whether the same sample was measured repeatedly
☐	☒	The statistical test(s) used AND whether they are one- or two-sided<br>*Only common tests should be described solely by name; describe more complex techniques in the Methods section.*
☒	☐	A description of all covariates tested
☐	☒	A description of any assumptions or corrections, such as tests of normality and adjustment for multiple comparisons
☐	☒	A full description of the statistical parameters including central tendency (e.g. means) or other basic estimates (e.g. regression coefficient) AND variation (e.g. standard deviation) or associated estimates of uncertainty (e.g. confidence intervals)
☐	☒	For null hypothesis testing, the test statistic (e.g. F, t, r) with confidence intervals, effect sizes, degrees of freedom and P value noted<br>*Give P values as exact values whenever suitable.*
☒	☐	For Bayesian analysis, information on the choice of priors and Markov chain Monte Carlo settings
☒	☐	For hierarchical and complex designs, identification of the appropriate level for tests and full reporting of outcomes
☒	☐	Estimates of effect sizes (e.g. Cohen's d, Pearson's r), indicating how they were calculated

Our web collection on statistics for biologists contains articles on many of the points above.

Software and code

Policy information about availability of computer code

Data collection	We used πDMD v1.0 for MD simulations. We used Leica microsystem software (Leica Application Suite X v5.0.2) for confocal image data collection.
Data analysis	The data analysis methods are clearly defined in the manuscript. We used and ImageJ/FiJi 1.53b open-source software for image analysis. We used Pymolv2.5, Modeller-9v14, VMD v1.9.3 for model building, simulation trajectory analysis and movie generation. We used GraphPad Prism 8v8.2.1, Microsoft Excel v16.49 for statistical analysis and plotting the graphs.

For manuscripts utilizing custom algorithms or software that are central to the research but not yet described in published literature, software must be made available to editors and reviewers. We strongly encourage code deposition in a community repository (e.g. GitHub). See the Nature Portfolio guidelines for submitting code & software for further information.

Data

Policy information about availability of data

All manuscripts must include a data availability statement. This statement should provide the following information, where applicable:

- Accession codes, unique identifiers, or web links for publicly available datasets
- A description of any restrictions on data availability
- For clinical datasets or third party data, please ensure that the statement adheres to our policy

We used RCSB PDB protein data bank to obtain 2J0J and 2V0U PDB structures (https://www.rcsb.org/structure/2J0J and https://www.rcsb.org/structure/2V0U). Plasmids harboring important genes used in this study are available from Addgene: uniRapR, FAK and LOV2 from Addgene plasmids #45381, #25928 and #87356, respectively The data that supports the findings of this paper are included in the Supplementary Material and Source data file. All other data are available from the corresponding author upon reasonable request. We will be depositing larger files such as Imaging data and simulations files in a public repository.

Field-specific reporting

Please select the one below that is the best fit for your research. If you are not sure, read the appropriate sections before making your selection.

[x] Life sciences   [ ] Behavioural & social sciences   [ ] Ecological, evolutionary & environmental sciences

For a reference copy of the document with all sections, see nature.com/documents/nr-reporting-summary-flat.pdf

Life sciences study design

All studies must disclose on these points even when the disclosure is negative.

Sample size	The sample size was not pre-calculated. For each experiment, sample sizes were chosen based on similar experiments reported in previous studies. The sample sizes are indicated in the figure captions. At least 3 biological replicates were performed for all the cases.
Data exclusions	No data were excluded from the analyses.
Replication	Each experiment was independently repeated at least three times with similar results.
Randomization	All the samples and controls were treated using the identical protocols. The detected cells or imaging views were randomly selected.
Blinding	Blinding was not relevant to our study, because any group allocation was not performed.

Reporting for specific materials, systems and methods

We require information from authors about some types of materials, experimental systems and methods used in many studies. Here, indicate whether each material, system or method listed is relevant to your study. If you are not sure if a list item applies to your research, read the appropriate section before selecting a response.

Materials & experimental systems

n/a	Involved in the study	
[ ]	[x]	Antibodies
[ ]	[x]	Eukaryotic cell lines
[x]	[ ]	Palaeontology and archaeology
[x]	[ ]	Animals and other organisms
[x]	[ ]	Human research participants
[x]	[ ]	Clinical data
[x]	[ ]	Dual use research of concern

Methods

n/a	Involved in the study	
[x]	[ ]	ChIP-seq
[x]	[ ]	Flow cytometry
[x]	[ ]	MRI-based neuroimaging

Antibodies

Antibodies used	Phospho-FAK (Y397) (Sigma-Aldrich, #ABT135), Phospho-paxillin (Y31) (Invitrogen, #44-720G), FAK (Santa Cruz Biotechnology, #SC557), Paxillin (Sigma-Aldrich, #SAB4502553), β-actin (Cell signaling, #8457). Horseradish peroxidase-linked secondary antibodies: Goat Anti-Mouse IgG Antibody, Peroxidase Conjugated (Sigma-Aldrich, #AP124P) and Goat anti-Rabbit IgG (H+L) Secondary Antibody, HRP conjugated (ThermoFisher Scientific, #31460).
Validation	All the antibodies are authenticated by manufacturer.<br>1. Phospho-FAK (Y397) (Sigma-Aldrich, #ABT135)- authenticated by western blot. https://www.sigmaaldrich.com/US/en/product/mm/abt135<br>2. Phospho-paxillin (Y31) (Invitrogen, #44-720G)- authenticated by western blot. https://www.thermofisher.com/antibody/product/Phospho-Paxillin-Tyr31-Antibody-Polyclonal/44-720G<br>3. FAK (Santa Cruz Biotechnology, #SC557),- authenticated by western blot. https://www.scbt.com/p/fak-antibody-d-1?requestFrom=search (https://www.scbt.com/p/fak-antibody-a-17?requestFrom=search)<br>4. Paxillin (Sigma-Aldrich, #SAB4502553)- authenticated by western blot. https://www.sigmaaldrich.com/US/en/product/sigma/sab4502553?context=product.<br>5. β-actin (Cell signaling, #8457)- authenticated by western blot. https://www.cellsignal.com/products/primary-antibodies/b-actin-d6a8-rabbit-mab/8457<br>6. Horseradish peroxidase-linked secondary antibodies: Goat Anti-Mouse IgG Antibody, Peroxidase Conjugated (Sigma-Aldrich, #AP124P)- authenticated by western blot. https://www.sigmaaldrich.com/US/en/product/mm/ap124p?context=product<br>7. Goat anti-Rabbit IgG (H+L) Secondary Antibody, HRP conjugated (ThermoFisher Scientific, #31460).- authenticated by western blot. https://www.thermofisher.com/antibody/product/Goat-anti-Rabbit-IgG-H-L-Secondary-Antibody-Polyclonal/31460

Eukaryotic cell lines

Policy information about cell lines

Cell line source(s)	HeLa (ATCC CCL-2), MDA-MB-231 (ATCC HTB-26), and FAK /- fibroblast cells (ATCC CRL-2644)
Authentication	All the cell lines were procured from ATCC and therefore, not authenticated. ATCC authenticates cell lines using STR analysis, according to the product specification.
Mycoplasma contamination	The cell lines were not tested for mycoplasma contamination.
Commonly misidentified lines (See ICLAC register)	No commonly misidentified cell lines were used
